# Both Full-Length and Protease-Cleaved Products of Osteopontin Are Elevated in Infectious Diseases

**DOI:** 10.3390/biomedicines9081006

**Published:** 2021-08-13

**Authors:** Toshio Hattori, Hiroko Iwasaki-Hozumi, Gaowa Bai, Haorile Chagan-Yasutan, Ashwnini Shete, Elizabeth Freda Telan, Atsushi Takahashi, Yugo Ashino, Takashi Matsuba

**Affiliations:** 1 Research Institute of Health and Welfare, Kibi International University, Takahashi 716-8508, Japan; hiro_ihz@kiui.ac.jp (H.I.-H.); gaowabai@kiui.ac.jp (G.B.); haorile@gjmyemail.gjmyy.cn (H.C.-Y.); atakah7@kiui.ac.jp (A.T.); 2 Mongolian Psychosomatic Medicine Department, International Mongolian Medicine Hospital of Inner Mongolia, Hohhot 010065, China; 3 ICMR-National AIDS Research Institute, 73 G-Block, MIDC, Bhosari, Pune 411026, India; ashete@nariindia.org; 4 STD AIDS Cooperative Central Laboratory, San Lazaro Hospital, Manila 1003, Philippines; betelan@yahoo.com; 5 Department of Respiratory Medicine, Sendai City Hospital, Sendai 982-8502, Japan; ashino-yug@hospital.city.sendai.jp; 6 Department of Animal Pharmaceutical Science, School of Pharmaceutical Science, Kyusyu University of Health and Welfare, Nobeoka 882-8508, Japan; matsubat@phoenix.ac.jp

**Keywords:** osteopontin, infectious disease, tuberculosis, adult T-cell leukemia, human immunodeficiency virus, dengue virus, hepatitis C virus, leptospirosis, proteases, matrix metalloproteinase

## Abstract

Circulating full-length osteopontin (FL-OPN) is elevated in plasma from patients with various infectious diseases, such as adult T-cell leukemia, *Mycobacterium tuberculosis* (TB), hepatitis virus infection, leptospirosis, acquired immune deficiency syndrome (AIDS), AIDS/TB, and coronavirus disease 2019 (COVID-19). Proteolysis of OPN by thrombin, matrix metalloproteases, caspase 8/3, cathepsin D, plasmin, and enterokinase generates various cleaved OPNs with a variety of bioactivities by binding to different target cells. Moreover, OPN is susceptible to gradual proteolysis. During inflammation, one of the cleaved fragments, N-terminal thrombin-cleaved OPN (trOPN or OPN-Arg^168^ [OPN-R]), induces dendritic cell (DC) adhesion. Further cleavage by carboxypeptidase B2 or carboxypeptidase N removes Arg^168^ from OPN-R to OPN-Leu^167^ (OPN-L). Consequently, OPN-L decreases DC adhesion. In particular, the differences in plasma level over time are observed between FL-OPN and its cleaved OPNs during inflammation. We found that the undefined OPN levels (mixture of FL-OPN and cleaved OPN) were elevated in plasma and reflected the pathology of TB and COVID-19 rather than FL-OPN. These infections are associated with elevated levels of various proteases. Inhibition of the cleavage or the activities of cleaved products may improve the outcome of the therapy. Research on the metabolism of OPN is expected to create new therapies against infectious diseases.

## 1. Introduction

**Figure 1:**
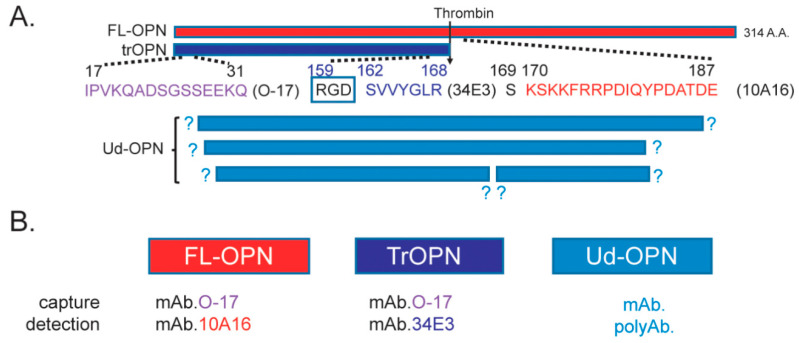
ELISA system employed to measure osteopontin (OPN). (**A**). Amino acid sequence of the epitope of the antibodies used for the assay. (**B**). Antibodies used for the ELISA kits. Abbreviations are FL-OPN, full-length OPN; trOPN, thrombin-cleaved OPN; and Ud-OPN, undefined OPN.

Osteopontin (OPN) is a phosphoprotein and is secreted by transformed cell lines from several mammalian species in the 58,000-Da molecular-weight range [1]. Subsequently, OPN was shown to be secreted by activated macrophages and T-lymphocytes abundantly. Furthermore, a cleaved 45 kDa product and a 70 kDa species secreted from T cells were identified [2]. It was also found that OPN belongs to matricellular proteins characterized by the direct binding to other matrix proteins, triggering of their specific surface receptors, and binding to proteases, growth factors, and cytokines (which modulates their activities). OPN also modulates several processes, like cell adhesion and migration, extracellular matrix (ECM) deposition, cell survival, and proliferation [3,4]. Detection of various forms of OPN influencing these functions was sought by an enzyme-linked immunosorbent assay (ELISA) system using antibodies against synthetic peptides [5]. OPN is known to be cleaved by various proteases, and, among them, thrombin cleavage maybe important, because they localize together in various inflammations (Figure 1) [6,7]. The cleaved fragments maintain OPN adhesive function and expose new active domains that may impart new activities. Thrombin cleavage leads to exposure of the SVVYGLR cryptic domain that induces cell adhesion and migration by binding to integrins α4 and α9 in vitro [8]. Using the combination of antibodies, namely, O-17 specific to the N-terminus of OPN (Ile^17^–Gln^31^) and 34E3 specific to N-terminal thrombin-cleaved OPN (trOPN) epitope (Ser^162^–Arg^168^), exposed by thrombin digestion, it was possible to measure trOPN [9]. Thereafter, plasma full-length OPN (FL-OPN) and trOPN levels were measured using a commercial ELISA kit (Immuno-Biological Laboratories, Gunma, Japan) and another kit (R&D Systems, Minneapolis, MN, USA) that detect both FL-OPN and the cleaved products, though their epitopes were undefined (Ud-OPN) (Figure 1) [10,11].

These studies enabled the development of new treatment based on metabolism of OPN. It was proposed that antibody against thrombin cryptic epitope of OPN inhibits metastasis as well as tumor growth in a mouse model of adult T-cell leukemia (ATL) [12] and attenuates liver inflammation in a non-alcoholic steatohepatitis mouse model [13]. Furthermore, new derivatives of brefelamide, aromatic amide isolated from the *Dictyostelium* cellular slime model, were reported to inhibit OPN synthesis in THP-1 cells [14]. In this review, we mention the biological activities of FL-OPN and its cleaved products and the summary of clinical studies measuring them and implicated novel roles of OPN in infectious diseases.

## 2. OPN Proteolysis and Bioactivities of Cleaved OPNs

**Figure 2:**
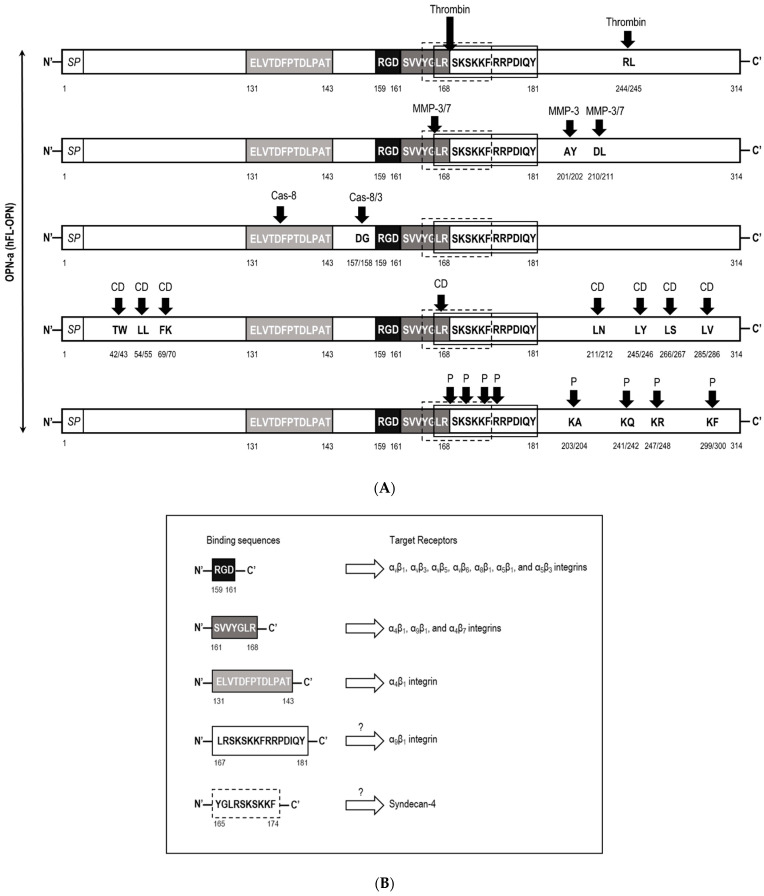
Identified protease cleavage sites in human FL-OPN (**A**,**B**). Schematic representation of cleavage sites of thrombin, MMPs, caspases, cathepsin D, and plasmin are separately indicated by black arrows. The amino acid sequences binding to different target receptors, including integrins and syndecan-4, are shown in black, gray, white, or dashed-line boxes (**A**). The target receptors for each binding sequence of OPN are listed (**B**). Abbreviations are OPN-a, a canonical isoform of human OPN among its splicing variants; Cas-,caspase; CD, cathepsin D; P, plasmin; and SP, signal peptides. “?”, unknown.

OPN undergoes numerous posttranslational modifications, such as serine/threonine phosphorylation, sulfation, glycosylation, glutamination, and proteolytic processing, which significantly contribute to the functions of OPN. Proteolytic processing either increases or reduces the ability of OPN to bind to target receptors. Thus, small differences in the cleavage pattern result in a substantial effect on the functions of OPN. To date, thrombin [15,16,17], matrix metalloproteinases (MMPs) [18,19,20], caspase-8/3 [21], plasmin [22], cathepsin D [22], and enterokinase [23] have been identified as proteases that cleave OPN (Figure 2). The fragments generated by their cleavage have a variety of bioactivities.

### 2.1. Binding Specificities and Cellular Functions of Thrombin-Cleaved OPN

Cleaved OPN was first identified during blood coagulation [15]. FL-OPN and thrombin are believed to be present together wherever the coagulation pathway is activated in inflammation, tumors, and wounds [24]. Thrombin cleavage sites were identified close to a highly conserved ^158^GRGDS^162^ [16,17,25,26]. In the sites, the RGD domain binds to integrin receptors, including α_v_β_1_, α_v_β_3_, α_v_β_5_, α_v_β_6_, α_8_β_1_, α_5_β_1_, and α_5_β_3_ [27,28,29,30,31,32,33,34,35]. In addition, α_4_β_1_, α_9_β_1_, and α_4_β_7_ integrins bind to the cryptic ^162^SVVYGLR^168^ sequence, which appears at Arg^168^-Ser^169^ by thrombin cleavage, in an RGD-independent manner [34,36,37,38,39,40,41,42,43]. The ^131^ELVTDFPTDLPAT^143^ domain has also been shown to bind to α_4_β_1_ [39]. Notably, another conserved sequence, ^165^YGLRSKSKKF^174^, includes the thrombin cleavage sites in mice and binds to heparin sulfate on syndecan-4, protecting OPN from cleavage by thrombin [44].

**Figure 3:**
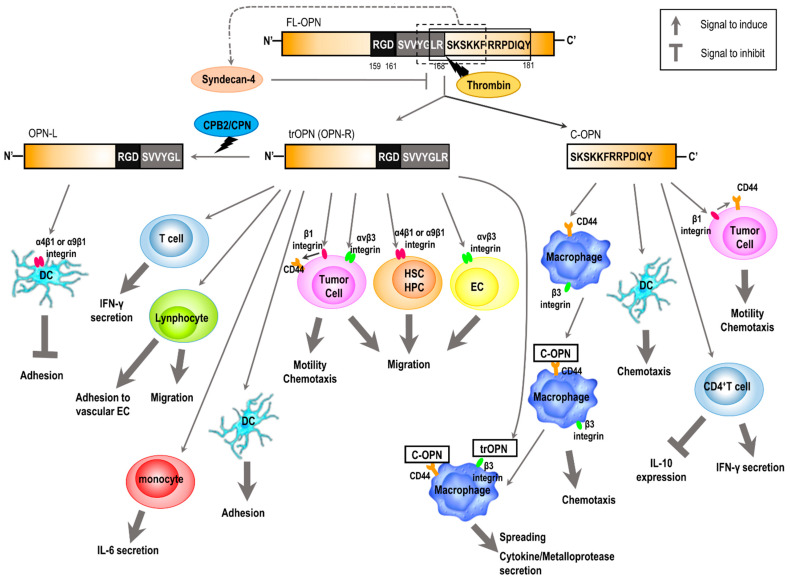
Cellular functions regulated by thrombin cleavage of OPN. The proposed mechanism regulated by thrombin cleavage of OPN. The trOPN (OPN-R) induces tumor cell migration, motility, and chemotaxis; IFN-γ secretion from T cells; and migration of HSC, HPC, and EC by binding to integrins. During the progression of inflammation, thrombin cleavage of OPN occurs and the cleaved fragments, trOPN (OPN-R), induce dendritic cell (DC) adhesion. OPN-L generated by CPB2 or CPN cleavage of trOPN (OPN-R) decreases DC adhesion by binding to α4β1 or α9β1 integrins. C-OPN induces DC chemotaxis. On the other hand, C-OPN induces tumor cell motility and chemotaxis via the interaction between β1 integrin and CD44. C-OPN also induces macrophage chemotaxis interacting with CD44 on macrophages leading to cellular attachment to trOPN via β3 integrin. The attachment of trOPN to macrophages leads to cell spreading, cytokine secretion, and release of metalloproteases. Abbreviations are C-OPN, thrombin-cleaved C-terminal OPN; OPN-R, OPN-Arg^168^; OPN-L, OPN-Leu^167^; CPB2, carboxypeptidase B2; CPN, carboxypeptidase N; DC, dendritic cell; HSC, hematopoietic stem cell; HPC, hematopoietic progenitor cell; EC, endothelial cell; and IFN-γ, interferon-γ.

The N- and C-terminal fragments of OPN, whose molecular weights are approximately 35 kDa and 25 kDa, respectively, are produced by thrombin cleavage. The fragments have a variety of functions (Figure 3) that differ from those of FL-OPN [45]. The N-terminal fragment, trOPN, enhances interferon-gamma (IFN-γ) secretion by T cells and stimulates hematopoietic stem cell (HSC) and hematopoietic progenitor cell (HPC) migration by binding to α_4_β_1_ and α_9_β_1_ integrins [46,47]. In addition, trOPN is a ligand for α_v_β_3_ integrin and promotes tumor cell migration higher than FL-OPN and other ligands of α_v_β_3_ integrin, such as fibrinogen and vitronectin [48]. The regulation of endothelial cell migration by vascular endothelial growth factor (VEGF) can be modulated by induction of thrombin-cleaved OPN, which was assumed to be the N-terminal fragment, and by α_v_β_3_ integrin [49]. The C-terminal fragment inhibits interleukin (IL)-10 secretion and stimulates cell–cell adhesion by interacting with CD44 isoforms [50,51,52]. Additionally, the interaction of OPN with CD44 was suggested to be mediated via β_1_ integrin and is not dependent on RGD sequence [53]. On the other hand, Shao et al. demonstrated that OPN and its thrombin-cleaved C-terminal fragment do not bind to CD44 and its variant form, CD44v6 [54]. Two other studies have implicated CD44 and CD44v6 as receptors for OPN in experiments, because anti-CD44 antibodies inhibited the effects of OPN interactions with cells [52,53]. However, the results of the two studies may indicate that the anti-CD44 antibodies directly inhibit cell migration do not block the OPN interaction with CD44 [54].

The cleavage of OPN by thrombin affects chemokine-induced migration of a dendritic cell (DC) [54]. A series of mechanisms were proposed. In the first, at the initiation of inflammation when DCs are being activated, proteoglycan-bound FL-OPN exerts its maximal potentiating effects of DC migration, which is induced by chemokine, mediated via the ^159^RGD^161^ and ^168^RSKSKKFRR^176^ sequences despite the presence of thrombin. Secondly, along with progression of inflammation and generation of thrombin and carboxypeptidase B2 (CPB2) at sites, the cleavage by thrombin occurs, followed by the induction of cell adhesion by exposing SVVYGLR and access to RGD in trOPN (OPN-Arg^168^ [OPN-R]) while reducing its effect on DC migration. Thirdly, further cleavage by CPB2 or carboxypeptidase N (CPN), which can remove Arg^168^ from OPN-R, inactivates SVVYGLR. This converts OPN-R to the N-terminal fragment 1-167 (OPN-Leu^167^ OPN-L) and decreases cell adhesion mediated by α_4_β_1_ and α_9_β_1_ integrins [55,56]. Fourthly, while thrombin cleavage disrupts ^168^RSKSKKFRR^176^ pro-chemotactic domain in FL-OPN, it releases the C-terminal fragment Ser^169^-Asn^314^, which shows substantial pro-chemotactic activity, which compensates for the loss of the pro-chemotactic activity in OPN-R and -L.

Both trOPN and thrombin-cleaved C-terminal OPN were proposed to regulate responses of neutrophil-independent macrophages, which are a part of the delayed healing processes [52]. OPN is secreted from activated T cells and the thrombin cleavage of OPN releases trOPN and the C-terminal fragments extracellularly. The C-terminal fragments induce chemotaxis of macrophages interacting with CD44 on macrophages, leading to cellular attachment to the trOPN via β_3_ integrin. The attachment of trOPN to macrophages leads to cell spreading and activation, including the induction of cytokine secretion and release of metalloproteases that were able to degrade the ECM.

### 2.2. Binding Specificities and Functions of MMP-Cleaved OPN

Different members of the MMP family are detected during injury and disease processes together with OPN [57]. MMP-3 (stromelysin-1), MMP-7 (matrilysin), MMP-2, and MMP-9 have been reported to cleave OPN [18,19,20]. A Gly^166^-Leu^167^ bond in the C-terminal to the RGD sequence is cleaved by both MMP-3 and -7 efficiently, and both also cleave OPN at the Asp^210^-Leu^211^ bond. Additionally, the cleavage site of only MMP-3 was found at Ala^201^-Tyr^202^ in OPN [18]. Cleavage by MMP-3 and -7 generates an N-terminal fragment, which contains the RGD sequence and also an SVVYG sequence of the SVVYGLR domain that is recognized by α_4_β_1_ and α_9_β_1_ integrins. However, neither the SVVYG sequence nor α_4_β_1_ plays a role in adhesion to the MMP-cleaved OPN. The anti-α_9_β_1_ antibody also did not inhibit additional cell binding to MMP-cleaved OPN. OPN and MMP-3 are expressed both in a temporal and cell-specific fashion during the progression of squamous cell carcinoma [58,59]. The ^152^LRSKSRSFQVSDEQY^166^ motif in the C-terminal fragment of MMP-3/7-cleaved mouse OPN, which corresponds to ^167^LRSKSKKFRRPDIQY^181^ in human OPN including different amino acids from mouse OPN, also binds to α_9_β_1_ integrin (Figure 2) and is involved in the occurrence of anti-type II collagen antibody-induced arthritis [60]. Expression patterns of OPN and MMP-3 were overlapped in the stroma during skin incisional wound healing [61] and those of OPN and MMP-7 were overlapped during involution of the postpartum uterus [62] in mice experiments. Higher expressions of OPN and MMP-7 are closely associated with the occurrence, progression, and prognosis of non-small cell lung cancer (NSCLC) [63].

An alternative splicing event promotes extracellular cleavage of OPN by MMP-9, and the RGD-independent region of the cleaved fragment avidly enhances hepatocellular carcinoma (HCC) cellular invasion [20]. Moreover, Leituner et al. found that the correlation of OPN and MMP-9 expression was found in adipose tissue from obese individuals, and increased levels of cleaved OPN were detected in the adipose tissue of these individuals [64]. The authors also showed that OPN cleavage by MMP-9 occurs in obesity and enhances the inflammatory and pro-diabetic activity of OPN in adipocytes. In patients with severe osteoarthritis, dysregulation of OPN, MMP-9, and metalloproteinase with thrombospondin motifs 4 (ADAMTS-4) and a disintegrin was demonstrated to be important for the pathogenesis, suggesting that MMP-9 and thrombin are induced in osteoarthritis. Both MMP-9 and thrombin can cleave OPN and consequently downregulate ADAMTS-4 [65]. MMP-9 can cleave OPN at least 30 cleavage sites, and the fragment generated by cleavage at Gly-Leu was shown to increase cardiac fibroblast migration in mice [66].

In EAE, cleavage of OPN by MMP-12 may occur and EAE disease activity might be modulated [67].

### 2.3. Binding Specificities and Functions of Caspase-Cleaved OPN

The molecular weights of the Caspase-8 and -3-cleaved products of OPN are different from the previously reported thrombin-cleaved products [21]. Caspase-8 cleaves OPN at both Asp^135^ and Asp^157^, and caspase-3 cleaves OPN at Asp^157^, which is localized in the N-terminal to the RGD sequence. OPN expression is rapidly increased during hypoxia or reoxygenation, which is associated with pathological conditions, such as myocardial ischemia/reperfusion injury, stroke, solid tumors, and inflammation [68,69] Subsequently, OPN is cleaved by caspase-8. This leads to inactivation of AKT signaling and activation of cell death signal via the cleaved fragment in tumor cells [21]. Notably, the C-terminal fragment generated by caspase-8 localizes in the nucleus, whereas the N-terminal fragment was found in the plasma membrane. It was also suggested that the C-terminal fragment may gain proapoptotic activity by modulating p53 in the nucleus, although the N-terminal fragment does not show any activity on cell death.

### 2.4. Binding Specificities and Functions of Plasmin and Cathepsin D-Cleaved OPN

Seven N-terminal fragments of OPN generated by proteolytic cleavage in the region of Leu^167^-Arg^175^ next to the RGD sequence were identified in human milk [22]. All fragments were generated by cleavage of the Leu^167^-Arg^168^, Arg^168^-Ser^169^, Ser^169^-Lys^170^, Lys^170^-Ser^171^, Ser^171^-Lys^172^, Lys^172^-Lys^173^, or Phe^174^-Arg^175^ bonds. The cleavage of Arg^168^-Ser^169^ matched the thrombin cleavage site for OPN, whereas the other six cleavages did not correspond to cleavage by thrombin or MMPs. Consequently, plasmin and cathepsin D, two endogenous proteases, were able to generate three of the seven N-terminal fragments. Plasmin, one of the principal proteases in milk, hydrolyzes Arg^168^-Ser^169^, which is the same peptide bond as thrombin, but mainly cleaves the Lys^170^-Ser^171^ bond. Another endogenous milk protease, cathepsin D, cleaves the Leu^167^-Arg^168^ bond. All of the cathepsin D- and plasmin-cleavage sites of OPN containing the three peptide bonds are shown in Figure 2.

Plasmin cleavage of OPN is not restricted to milk because the cleaved fragment of OPN by plasmin was also identified in urine [22]. Plasmin-cleaved OPN induces cell adhesion mediated by α_v_β_3_ and α_5_β_1_ integrins, indicating that plasmin could regulate OPN activity. Notably, modifications, such as phosphorylation, in the C-terminal cleaved OPN significantly reduced the adhesion of cells to OPN via α_v_β_3_ integrin, whereas the N-terminus did not influence the binding [70]. Inhibitory binding could be restored by proteolytic removal of the C-terminus by plasmin and thrombin, rather than by better exposure of the RGD sequence.

### 2.5. Gradual Proteolysis of OPN Regulating Cellular Functions

OPN was suggested to be susceptible to gradual proteolysis by different types of proteases and, thus, each gradually cleaved OPN is assumed to play different roles in regulating cellular functions. Recently, we characterized various forms of OPN, including FL-OPN, and several types of cleaved OPN in phorbol 12-myristate 13-acetate (PMA)-differentiated THP-1 macrophages and identified a novel, small-sized fragment (MW 18 kDa) generated by proteolysis using antibodies against distinct protein epitopes. [71]. MMP-9 could be induced in THP-1 cells treated with PMA [72]. In *Mycobacterium tuberculosis* (MTB)-infected THP-1 cells, MMP-9 was increased by microRNA-206 [73] and the expression of lysosomal cathepsin B and D was upregulated [74]. Therefore, the gradual cleavage of OPN by several types of proteases can occur during differentiation and inflammation of THP-1 cells. The bioactivities of each cleaved fragment need to be further clarified.

### 2.6. Different Expression and Localization between FL-OPN and Its Cleaved OPNs

The expression of FL-OPN, trOPN, and Ud-OPN was decreased in PMA-differentiated THP-1 macrophages followed by Bacillus Calmette-Guérin (BCG) infection. In contrast, BCG infection enhanced OPN proteolysis and increased the extracellular localization of the cleaved OPNs [71]. The results suggest that the proteolysis of OPN may occur within the activated lysosome of PMA-differentiated THP-1 macrophages after BCG infection, with the cleaved OPN fragments released extracellularly.

### 2.7. The Roles of Thrombin-Cleaved OPN in the Pathogenesis of Inflammatory Diseases

OPN and thrombin are highly expressed during inflammation. Many studies have characterized the functions of trOPN, especially among the several cleaved forms of OPN, for the pathogenesis of a wide range of inflammatory diseases. Both the roles of trOPN and thrombin-cleaved C-terminal OPN have been suggested in cancer because of the expression of both cleaved OPNs and activated thrombin in tumor cells [75,76,77]. In a study of malignant glioblastoma (GBM), the levels of trOPN (OPN-R) and OPN-L were markedly elevated in both tissue and cerebrospinal fluid (CSF) of malignant GBM patients compared to systemic cancer and non-cancer patients [76]. OPN and its cleaved forms induce cell migration and confer resistance to apoptosis in GBM cells.

FL-OPN was suggested to be expressed in the synovial fluid of patients with rheumatoid arthritis (RA) at similar levels to those with osteoarthritis, whereas the levels of trOPN in RA synovial fluid are approximately 30-fold higher than in those with osteoarthritis and correlate with disease severity [78]. Similarly, the levels of cleaved OPNs are markedly increased in the synovial fluid of patients with RA, but not in osteoarthritis or psoriatic arthritis [56].

Thrombin activity increases with the progression of neuroinflammation and is detectable in demyelinating lesions where OPN is also present at high levels [79,80]. Administration of a thrombin inhibitor decreased clinical severity, demyelination, and secretion of Th1- and Th17-type cytokines in experimental autoimmune encephalomyelitis (EAE) [81,82]. Thrombin-mediated cleavage of OPN also plays a key role in multiple sclerosis relapse by exerting a dual effect [83]. On the one hand, thrombin-cleaved OPNs may be crucial in the homing of autoreactive lymphocytes in the central nervous system lesions. In this setting, trOPN increases production of IL-17 involved in breaking the blood–brain barrier and stimulating lymphocyte migration, whereas the C-terminal fragment increases lymphocyte adhesion to vascular endothelial cells. On the other hand, the cleaved OPNs may support inflammation, in which the N-terminal fragment induces secretion of IL-6 from monocytes and inhibits IL-10 production in CD4^+^ T cells, increasing the secretion of IFN-γ from CD4^+^ T cells.

Thrombin-cleaved OPN is involved in the progression of liver fibrosis in vitro and in vivo and induces the activation of hepatic stellate cells by increasing the expression of α4 and α9 integrins via mitogen-activated protein kinase (MAPK) and nuclear factor-kappa B (NF-κB) signaling pathways [84]. It has also been suggested that trOPN in urine reflects renal inflammation [85]. The urine level of trOPN was significantly higher in patients with lupus nephritis than in healthy controls. Moreover, patients with overt proteinuria (urine protein/creatinine ratio > 0.5) had significantly higher concentrations of urine trOPN than those with minimal proteinuria (urine protein/creatinine ratio < 0.5) and diabetic nephropathy patients with overt proteinuria (urine protein/creatinine ratio > 0.5).

## 3. Immunological Activities of OPN in Infectious Diseases

The adaptive immunity characterized by the generation of T helper type (Th)1 cells and cytotoxic T lymphocytes is critical for protecting hosts from colonization by intracellular pathogens. To induce a rapid and effective Th1-mediated immunity, cytokine release is dominated by the production of IL-12. OPN has been suggested to promote Th1 response through the production of IL-12 derived from macrophages, monocytes, and DCs [86,87,88]. Several disease models in OPN-deficient mice have implicated the role of OPN as a Th1-driving cytokine.

Ashkar et al. demonstrated that OPN-deficient mice revealed a severely impaired Th1-mediated immunity against herpes simplex virus type 1 and *Listeria monocytogenes*. OPN-mediated Th1 immunity was stimulated via the induction of IL-12 from macrophages by the interaction between the amino-terminal portion of OPN and α_v_β_3_ integrin, as well as the suppression of anti-inflammatory cytokine IL-10 by the interaction of OPN with CD44 [86]. Another group reported that OPN-deficient mice died after *Plasmodium chabaudi* infection, while wild-type (WT) mice had self-limiting infections and produced significantly smaller amounts of IL-12 and IFN-γ than did WT mice [89]. Furthermore, various toxins and microbial components induce the secretion of IL-12 from DCs. IL-12 produced by DCs induces the differentiation of CD4^+^ T cells toward Th1 cells and stimulates the secretion of IFN-γ from unsensitized natural killer (NK) and T cells [90]. Renkle et al. demonstrated that OPN stimulates IL-12 secretion from DCs, promoting IFN-γ production by T cells and, consequently, OPN-activated DCs to promote Th1 polarization of naïve T cells [88].

However, contrary to the above results, another report described that viral clearance, lung inflammation, and recruitment of effector T cells to the lung were unaffected in OPN-deficient mice after influenza infection and that OPN-deficient mice mounted an unimpaired immune response to *Listeria monocytogenes*. OPN produced by either CD4^+^ T cells or DCs did not stimulate naïve CD4^+^ T cells or induce its differentiation toward IFN-γ-producing Th1 cells [91]. Interestingly, the model of over-expressed OPN in mice and cells that immunized heat-killed *Listeria monocytogenes* (HKLM) indicated that a mature DC migration from antigen entry sites to lymph node is suppressed by over-expressed OPN because HKLM-induced CCR7 expression on DCs was inhibited by OPN, suggesting that an OPN-mediated negative feedback can contribute to impaired T cell immunity through the regulation of DC migration [92].

OPN was found to enhance the release of Th1 and Th17 cytokines, which exert protective functions against infection with *Trypanosoma cruzi*, an etiological agent of Chagas disease. The release of Th1 and Th17 cytokines is preferentially regulated through the interaction of OPN with α_v_β_3_ integrin accompanied with the inhibition of IL-10 production mostly depending on the interaction of OPN with CD44 [93].

OPN has been reported to regulate the replication of human immunodeficiency virus (HIV)-1 and hepatitis C virus (HCV). Knockdown of OPN in macrophages significantly suppressed HIV-1 replication in contrast to other intracellular pathogens. HIV infectivity and replication were promoted by ectopic expression of OPN in TZM-bl cells [94]. During HCV infection, endogenous OPN was involved in HCV replication and assembly through the association of OPN with lipid droplets along with HCV nonstructural (NS) and core proteins in endoplasmic reticulum. Secreted OPN bound to α_v_β_3_ integrin and CD44 regulate HCV replication and assembly through focal adhesion kinase-mediated expression of HCV NS and core proteins [95]. Thus, the clarification of the dynamics of OPN metabolism in distinct infectious diseases is essential for understanding the immune system and contributing to the treatment of a wide range of inflammatory diseases.

## 4. OPN in Infectious Diseases

### 4.1. ATL

ATL is caused by human retroviruses, human T-cell lymphotropic virus type 1 (HTLV-1). HTLV-1 is present worldwide, and it is interesting that high endemic clusters are often located near areas where the virus is nearly absent. HTLV-1 is mainly endemic in the southwestern part of Japan, sub-Saharan Africa, South America, the Caribbean area, and foci in the Middle East and Austral-Melanesia. The total number of HTLV-1 carriers is estimated to be 10–20 million [96]. HTLV-1 Tax viral protein activates NF-κB and autophagy pathways that favor viral replication and T-cell transformation [97]. It was reported that the OPN gene is trans-activated by Tax protein of HTLV-1, and the phosphoinositide 3-kinase (PI3K)/AKT pathway is involved in Tax-mediated transactivation, because Tax-induced OPN activation was abrogated by treatment with LY294002 (PI3K inhibitor) or co-transfection with AKT siRNA [98]. The gene encoding CD44 is also one of the downstream target genes of NF-κB signaling aberrantly activated by Tax [99]. It is of note that expression of both OPN and its receptor CD44 is enhanced by Tax-induced NF-κB signaling, indicating these molecules are integrated into a fate-determining cellular program. In patients with ATL, a marked elevation of FL-OPN (*p* = 3.6 × 10^−6^) and soluble CD44 (*p* < 0.001) were observed in plasma, and they were significantly related to each other (*p* < 0.002) [100]. The levels of plasma OPN were the highest in acute ATL, followed by lymphoma and chronic ATL (Table 1). Moreover, the levels in acute ATL or acute lymphoma were significantly higher than those in chronic ATL or chronic and smoldering ATL, respectively. In addition, they were significantly associated with the performance status, total number of involved lesions, and lactic dehydrogenase, and inversely with lymphocyte count (*p* < 0.01) These findings indicate that OPN reflects the severity of ATL (Table 2), and it should be noted the levels are also an independent prognostic factor in acute myeloid leukemia [101]. In lymph nodes and skin, immunohistochemically staining using anti-OPN and anti-CD44 antibodies disclosed that the expression of both OPN and CD44 was weak/moderate in ATL cells but moderate/strong in infiltrated macrophages [100]. Cancer cells can upregulate OPN production in macrophages and the secreted OPN plays a role in enhancing the clonogenicity of cancer cells [102]. In a xenograft mouse model, fibroblast-derived OPN plays a role in the growth of transplanted human ATL, and the antibodies against OPN suppressed tumor growth [12]. The ability of CD44 to bind to extracellular ligands such as OPN allows for cellular anchoring and activation of specific signaling pathways and MMP. MMP-7 expression was identified in HTLV-1-infected T-cell lines, peripheral blood ATL cells, and ATL cells in lymph nodes, but not in uninfected T-cell lines or normal peripheral blood mononuclear cells. MMP-7 expression was induced following infection of a human T-cell line with HTLV-1, and specifically by the viral protein Tax [103].

### 4.2. Tuberculosis (TB)

TB is the leading cause of death among infectious diseases. Simple and sensitive tests for diagnosing and monitoring TB are lacking. OPN is a promising candidate for diagnosis and assessments of the severity of illness and response to TB treatment. OPN is a part of the Th1-type immune response, which is important in protective immunity against TB. IL-12 and IFN-γ associated with Th1 immunity are dependent on OPN secretion by peripheral blood mononuclear cells infected with BCG [104]. OPN can be a macrophage chemoattractant and was found to be present in tuberculous granulomas, suggesting its involvement in granuloma formation [118]. Although granulomas contain the spread of mycobacteria in TB, the role of OPN in conferring protective immunity in TB is controversial. More severe mycobacterial infections have been characterized by heavier loads and delayed clearance of bacteria in OPN-deficient mice [119]. In contrast, lower pulmonary bacterial loads and lung inflammation have also been reported during the late phase of TB, conferring a modest survival advantage to OPN-deficient mice [120].

The plasma levels of FL-OPN were significantly higher (*p* < 0.0001) in patients with TB than in healthy controls (Table 1) [104,105,106,118]. Higher levels were found in acquired immune deficiency syndrome (AIDS) associated with TB (AIDS/TB) [106]. Ud-OPN levels were also significantly higher in patients with AIDS/TB and TB [105,106,114]. Although the OPN levels were significantly higher in human immunodeficiency virus (HIV)-negative pulmonary TB (PTB) patients than in those with latent TB, the same trend was not observed in HIV-infected patients, indicating its limitation in detecting TB development in HIV infection [114]. The levels decreased significantly after successful TB treatment, as indicated by clinical improvement or sputum smear conversion, suggesting its potential to monitor the effect of treatment [104,121].

In our study, the plasma levels of FL-OPN and Ud-OPN were measured simultaneously. FL-OPN and Ud-OPN indicated a high area-under-the-curve (AUC) value between normal versus TB (AUC: around 0.99) and normal versus AIDS/TB (AUC: around 0.97), suggesting that FL-OPN and Ud-OPN can discriminate normal and TB as well as normal and AIDS/TB. It is of note that the FL-OPN levels did not show any significant differences between multidrug-resistant (MDR) TB and non-MDR TB, whereas Ud-OPN was significantly (*p* = 0.0227) lower in MDR TB compared with non-MDR TB, suggesting that FL-OPN and Ud-OPN reflect different pathologies of TB [106].

Ud-OPN and IL-6 levels were higher in patients with severe chest X-ray grade, indicating their association with disease severity, and were positively correlated with each other [114]. The plasma levels of Ud-OPN were significantly higher in patients with severe PTB in which lesions involved four or more zones without cavities or more than three zones with cavities, as compared with those with non-severe PTB (Table 2).

In TB patients, Spearman’s correlation analysis revealed a significant correlation between plasma levels of OPN and soluble CD44. The levels of both OPN and sCD44 were associated with lymphocytopenia and the levels of C-reactive protein (CRP), IL-8, and IFN-γ induced protein 10 (IP-10). The negative association of FL-OPN with esat-6 specific memory T cell numbers indicate that OPN is involved in migration of lymphocytes to granuloma [122].

An in vitro study showed that many genes in THP-1 cells were upregulated 6 to 12 h after MTB infection. Most of these genes encode proteins involved in cell migration and homing, including the chemokines IL-8, OPN, monocyte chemotactic protein-1 (MCP-1), and macrophage inflammatory protein-1α (MIP-1α) [123].

Systemic levels of MMP-1, 8, 9, and 12 were significantly higher in patients with PTB than in those with extrapulmonary TB (EPTB) or latent TB (LTB), and in healthy controls [124]. Recent findings also showed that the levels of MMP9 and MMP12 in bronchoalveolar lavage were increased in PTB patients who smoked. Between-group analysis showed that the frequency of M1 macrophages was higher in non-smoker PTB patients, while more M2 macrophages were found in smokers without PTB than in non-smokers [125].

### 4.3. Acquired Immune Deficiency Sndrome (AIDS)

The increased levels of OPN in plasma and CSF were demonstrated and OPN is a marker of cerebral dysfunction of antiretrovirus therapy (ART) era [126]. Elevated levels of FL-OPN [127] and both FL- and Ud-OPN [106] in AIDS patients were reported. The persistent elevation of FL-OPN during ART was described and it was claimed that it may be useful to monitor systemic inflammation in ART-treated AIDS patients [127]. Another study also proposed that a proliferation-inducing ligand (APRIL) and B cell-activating factor (BAF), tumor necrosis factor (TNF)-receptor 1(R1), CD163, and OPN were biomarkers for likely poor immune recovery [128].

Detailed analysis of brain tissues and CSF showed elevated levels of OPN in HIV-associated dementia, and it was implicated that OPN stimulates HIV-1 replication in the brain [94]. Recently it was shown that OPN levels showed significant negative correlation with CD4 counts (r = −0.345) and showed significant positive correlation with viral loads (r = 0.334) of the HIV-infected patients, indicating that systemic replication might also be enhanced by OPN [114].

The plasma FL-OPN and Ud-OPN of AIDS patients were significantly elevated compared with healthy control (Table 1). In AIDS/TB, Ud-OPN levels were higher than HIV single infection [114] but the FL-OPN and Ud-OPN levels of AIDS/TB indicated no difference from those of AIDS or TB [106].

In HIV-1-infected individuals, the protein and enzymatic activities of MMP2 and MMP9 were increased in peripheral blood. The levels were more increased in HIV-associated neurocognitive disorders (HAND). In postmortem examination, the levels were also elevated in brain tissue of patients with HAND [129]. Similarly, in postmortem brain tissue, perineuronal nets (PNNs)-degrading enzyme was increased in HAND and OPN was abundant in the HIV-infected brain [130,131].

### 4.4. Hepatitis Virus Infection

An estimated 257 million people (3.5% of the world’s population) are chronically infected with hepatitis B virus (HBV) and 71 million (1% of the world’s population) with HCV. IL-17 and FL-OPN levels were significantly increased in patients with chronic hepatitis B. In addition, OPN markedly induced IL-17 expression in leukocytes in both humans and mice. β3 integrin, an OPN receptor, is reported to be critically involved in the induction of IL-17 production by OPN [107].

HBV-associated acute-on-chronic liver failure (ACLF) is a syndrome with a high rate of short-term mortality. It is important to identify patients at high risk of mortality. The levels of Ud-OPN in the prediction of 90-day mortality in patients with ACLF was evaluated [108]. Serum OPN levels were significantly higher in HBV-ACLF patients (*n* = 54) than in those with acute exacerbation of chronic hepatitis B (CHB, *n* = 20) and healthy controls (*n* = 20, both *p* < 0.01) (Table 1). Furthermore, the serum levels were higher in those patients who succumbed to HBV-ACLF than in surviving patients (*p* < 0.05). The collective findings indicate that the serum OPN level may be an independent risk factor associated with HBV-ACLF prognosis. OPN may be a predictor of the prognosis of patients with HBV-ACLF. However, the OPN levels of normal controls were significantly higher than those of other diseases, and only one study used serum instead of plasma [108]. It should be determined whether serum used for the assay could be the reason for the high titers, because clot formation of blood to collect the serum activates proteases, such as thrombin, and causes structural changes in OPN. The significance of OPN expression in HBV-induced liver cirrhosis (LC) was evaluated. In LC patients, plasma OPN levels were significantly higher than those in HBV-infected patients (LC, 4.52 [3.15–6.43], *p* < 0.001) [109]. Both HBV and HCV are major causes of HCC in liver cancer [132]. In one study, plasma OPN levels at baseline were significantly higher (67.4 ng/mL, *n* = 100; *p* < 0.001) in HCC cases than in controls (53.7 ng/mL, *n* = 194) [110]. A statistically significant positive association was observed between circulating OPN levels and HCC risk. Within 2 years of diagnosis, the combination of OPN and alpha-fetoprotein (AFP) best predicted HCC development, suggesting that measuring OPN and AFP could identify high-risk groups independently of a liver disease [110].

The involvement of secreted OPN in liver dysfunction is interesting. Notably, human recombinant OPN upregulates MMP-2 through the stromal cell-derived factor 1 alpha/C-X-C chemokine receptor type 4 axis, which is mediated by binding to integrin α_(v)_β_3_ and CD44v6. The PI3K/AKT and c-Jun N-terminal kinase pathways are activated in HepG2 and SMMC7721 cells [133]. Additionally, HBV infection results in altered membrane CD100 (mCD100) expression and serum-soluble CD100 (sCD100) levels. The sCD100 can increase the CTL response against HBV and accelerate HBV clearance. Shedding of CD100 by mice and sCD100 formation are mediated by MMP2 and MMP9 [134].

### 4.5. OPN in Tropical Neglected Diseases

#### 4.5.1. Dengue

Tropical areas are sites of various tropical infectious diseases. A metrological analysis correlated epidemic of dengue and leptospirosis with rainfall and with relative humidity and temperature in the Philippines [135]. Dengue is an increasing global public health threat. Four dengue virus types (DENV1–4) are now co-circulating in most dengue endemic areas. Population growth, an expansion of areas hospitable for the *Aedes* mosquito species, and the ease of travel have all contributed to a steady rise in dengue infections and disease. Dengue is common in more than 100 countries worldwide. Each year, up to 400 million people acquire dengue. Approximately 100 million people are infected and 22,000 die from severe dengue. The regions that are most seriously affected by outbreaks are the Americas, South/Southeast Asia, and the Western Pacific. Asia represents approximately 70% of the global burden of disease [136] (https://www.cdc.gov/dengue, accessed on 1 July 2021). DENV infections are categorized into three groups: undifferentiated fever, dengue fever (DF), and dengue hemorrhagic fever (DHF) [137]. DHF is further classified into four severity grades, the most severe of which is dengue shock syndrome [138].

**Figure 4:**
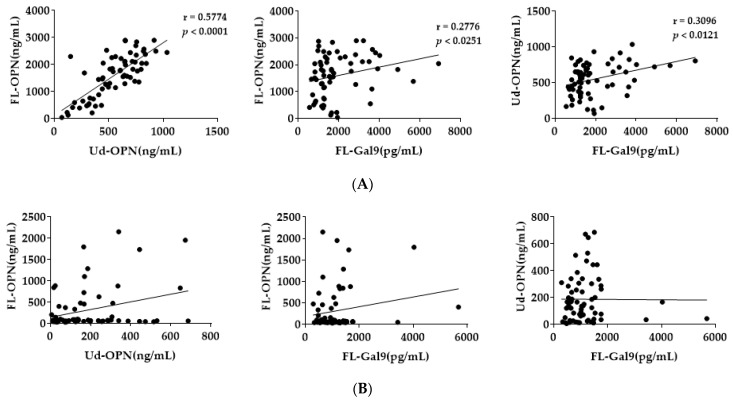
Correlations of FL-OPN, Ud-OPN, and full-length Galectin-9 (FL-Gal9) in dengue patients in the critical phase (**A**) and recovery phase (**B**). The data are from references [9,142].

We analyzed plasma from patients with DF and DHF using three different ELISA kits as described in Figure 1. Levels of FL-OPN, trOPN, Ud-OPN, D-dimer, thrombin anti-thrombin complex (TAT), and thrombomodulin were significantly elevated in the critical phase in both the DF and DHF groups compared with healthy controls (Table 1). During the recovery phase, FL-OPN levels declined, while trOPN levels increased dramatically in both the DF and DHF groups, indicating that cleavage of OPN occurs during the recovery phase. FL-OPN levels were directly correlated with D-dimer and ferritin levels, while trOPN levels were associated with platelet counts, TAT levels, and viral RNA load. A Spearman rank correlation coefficient analysis revealed a significant correlation between FL-OPN, Ud-OPN, and FL-Galectin-9 (Gal-9) during the critical phase (Figure 4A). However, no correlation was evident during the recovery phase, indicating that the ELISA kit to detect Ud-OPN measured not only FL-OPN but also the cleaved forms, but could not differentiate between them (Figure 4) [11].

An increase in circulating MMP9, MMP12, and MMP13 levels was detected in plasma from DF patients, whereas MMP2 levels did not present relevant changes when compared to healthy controls. Moreover, the MMP9 median level was higher in severe DF than in mild DF [139]. In another study, however, MMP2 was implicated as a potential biomarker for plasma leakage associated with dengue shock syndrome [140]. Furthermore, non-structural protein 1 (NS1) exposure of THP-1 monocytes resulted in altered cell morphology and activated them for release of proteins in 24 h. Expression of MMP-2, MMP-8, MMP-9, and MMP-14 genes was upregulated by NS1 exposure [141].

#### 4.5.2. Leptospirosis

Leptospirosis is a neglected zoonotic disease with a global distribution. It is endemic mainly in countries with humid subtropical or tropical climates, and has epidemic potential [143]. There are an estimated 1.03 million cases per year, resulting in 2.9 million disability-adjusted life years, with the highest burden in resource-poor tropical countries [144].

Leptospirosis patients display significantly higher levels of plasma FL-OPN (*p* < 0.0001) and trOPN (*p* < 0.01) compared to healthy controls (Table 1). FL-OPN levels have been significantly correlated with the levels of serum cystatin C (CyC) (r = 0.41, *p* < 0.0001), urine trOPN/creatinine (Cr) (r = 0.23, *p* < 0.05), urine N-acetyl-β-d-glucosidase (NAG)/Cr (r = 0.35, *p* < 0.001), urine clusterin/Cr (r = 0.33, *p* < 0.05), urine CyC/Cr (r = 0.33, *p* < 0.05), and serum Cr (r = 0.28, *p* < 0.01). These findings indicate the involvement of OPN in kidney injury. A significant increase in urine NAG/Cr (*p* < 0.05) levels in leptospirosis patients has been described previously, and an increased urine NAG level is a highly specific marker of proximal tubular disease [111]. OPN may be involved in tubular dysfunction and is released by infiltrating inflammatory macrophages in tubules [145]. Leptospires endowed with plasminogen (PLG) or plasmin can promote the transcriptional upregulation of MMP-9. Serum samples from patients with confirmed leptospirosis showed higher levels of PLG activators and total MMP-9 than serum samples from healthy subjects. It has also been suggested that this stimulated proteolytic activity occurs at the early stage of the disease and is involved in bacterial cell penetration of endothelial cells [141]. Differential diagnosis of DF from leptospirosis is often difficult because both diseases are acute febrile illnesses and occur after disasters, such as floods [135]. Leptospirosis is often misdiagnosed as DF and is underdiagnosed in endemic regions [146]. Furthermore, both diseases are accompanied by cytokine storms. Both proinflammatory and anti-inflammatory cytokines and chemokines are induced during the progression of DENV infection, suggesting that multifunctional mediators are involved in the associated pathogenesis [142,147,148].

The involvement of a cytokine storm and subsequent immunoparalysis in the development of severe leptospirosis in susceptible hosts has been demonstrated. The potential contribution of major proinflammatory cytokines in the development of tissue lesions and systemic inflammatory response, as well as the role of anti-inflammatory cytokines in contributing to the onset of a deleterious immunosuppressive cascade, still needs to be examined [149].

#### 4.5.3. Melioidosis

A causative agent of melioidosis is the aerobic, gram-negative, soil-dwelling bacillus *Burkholderia* (*B.*) *pseudomallei* and causes severe sepsis in approximately 46 countries in Southeast Asia and Northern Australia [150]. Current estimates suggest that 165,000 cases of melioidosis result in 89,000 deaths worldwide per year [151]. Plasma OPN levels in patients showed approximately 70-fold higher than healthy controls (Table 1) and non-survivors showed higher OPN levels than survivors on admission, indicating the levels can reflect the severity for melioidosis (Table 2). Using OPN-deficient mice models, OPN was found to contribute to lung inflammation and mortality [112]. It is known that Neutrophils kill up to 90% of intracellular *B. pseudomallei* organisms and neutrophil extracellular traps of neutrophils promote indirect generation of the host cytokine response [112]. The analysis of blood transcriptome showed that 50 were commonly up-regulated in all mouse models and the human disease of the 118 upregulated genes, including Arginase-1 and cytokine genes, as well as MMPs and TLRs [152].

#### 4.5.4. Parasite Infection

##### Human African Trypanosomiasis (HAT)

The numbers of reported cases of HAT, or sleeping sickness, were 50,000 new cases each year in the last decade of the 20th century [153]. When patients with a white blood cell (WBC) count of less than 5 WBC/μL and no trypanosomes were classified as S1 and those with more than 5 WBC/μL and/or trypanosomes in the CSF as S2 patients, the levels of OPN and β-2-microglobulin in CSF significantly discriminated between S1 and S2 patients with high sensitivity for 100% and 91% specificity, respectively, and were highly correlated with WBC counts. The CSF levels of OPN were significantly increased in patients with parasites in CSF than those without parasites in CSF. Furthermore, the levels significantly discriminated between patients without neurological signs, with moderate neurological signs, and severe neurological signs, and were higher as the severity of neurological signs were elevated, therefore suggesting that the OPN levels in CSF can reflect the severity for HAT (Table 2) [117]. The examination of CSF from patients with S1 and S2 also showed that ICAM-1 and MMP-9 alone or in combination are significant staging markers of HAT [154].

##### Schistosomiasis

Schistosomiasis is caused by *Schistosoma* spp. flatworms that affects severe diseases in over 200 million people from 76 countries and territories in tropical areas. More people may be at risk due to the increases in population in endemic areas [155]. It is important to study when it was eradicated. The last case of *S. japonicum* in Japan was found in 1977 and infected snails were last detected in 1982 [156]. The clinical manifestations generally cluster into three distinct forms of the disease: acute, hepatointestinal, and hepatosplenic schistosomiasis. Serum OPN levels of 28 patients with acute schistosomiasis were investigated and their circulating levels of OPN in the serum (*p* = 0.0001) were increased (Table 1). The increase begins at 5–6 weeks post-infection and peaked 7–11 weeks post-infection. The symptoms start to disappear 12 weeks after infection and circulating OPN levels start to fall. It was suggested that serum OPN could be a good biomarker to diagnose symptomatic acute schistosomiasis [113]. It was also found that macrophages are one of the major sources of OPN in the early phases in patients with hepatointestinal schistosomiasis, while bile ducts are the main producers of OPN in patients with hepatosplenic disease [157]. Significant upregulated expression of proinflammatory cytokines (IL-1α, IL-1β, and IL-8) and chemokines (CCL3, CCL4, and CXCL2) in neutrophils after 4 h in vitro stimulation with *S. japonicum* eggs was also found. Successively stimulated neutrophils release mitochondrial DNA and produce MMP-9 [158].

### 4.6. Coronavirus Disease 2019 (COVID-19)

The situation becomes more complex with COVID-19 epidemics, another acute febrile illness. Severe acute respiratory syndrome coronavirus 2 (SARS-CoV-2) has caused an ongoing pandemic of COVID-19 with more than 200 million cases and more than 4.25 million deaths as of the end of 5 August 2021 (https://coronavirus.jhu.edu/map.html, accessed on 6 August 2021). The severity of the infection is highly variable, ranging from asymptomatic infections to mild cold symptoms, to severe pneumonia, to respiratory failure requiring mechanical ventilation, and to death from multiple organ failure [159].

It is important to know how DENV infection affects SARS-CoV-2 infection, and vice versa. This is because serological cross-reaction was reported in two patients in Singapore with false-positive results from rapid serological testing for dengue, who were later confirmed to be infected with SARS-CoV-2 [160]. Whether previous contact with endemic infectious diseases, such as symptomatic dengue, might alter the prognosis of COVID-19 was investigated. Patients without previous dengue had a higher risk of death (hazard ratio: 0.44; 95% confidence interval: 0.22 to 0.89; *p* = 0.023) at the 60-day follow-up. These findings have raised the possibility that dengue may induce immunological protection against SARS-CoV-2 [161]. 

Contrary to the cross-protection hypothesis, prior DENV infection was associated with twice the risk of clinically apparent COVID-19 upon SARS-CoV-2 infection. The higher risk of clinically apparent COVID-19 among individuals with prior dengue has important health implications for communities sequentially exposed to DENV and SARS-CoV-2 [162]. Epidemiological studies have shown a significant decrease in DF cases and a contrasting increase in leptospirosis cases in the second quarter of 2020 compared with 2019 in Sri Lanka [163]. DF transmission is closely related to the movement of people, which was restricted during the COVID-19 pandemic, not so much because of the disease, but because of the lockdown restrictions. Leptospirosis is a disease that arises from livelihood exposure in peri-domestic environments. It is more likely to continue its usual cycle of infection and may increase in prevalence, despite the lockdown restrictions. It is difficult to distinguish non-severe cases of dengue from COVID-19 in the context of co-epidemics. A cohort study of co-epidemic areas described that non-severe dengue was more symptomatic than mild to moderate COVID-19. Body ache, headache, and retro-orbital pain were indicative of dengue, whereas contact with a COVID-19-positive case, anosmia, delayed presentation (>3 days post-symptom onset), and the absence of active smoking were indicative of COVID-19. Based on the study, basic clinical and epidemiological indicators may help distinguish COVID-19 and dengue from each other and other febrile illnesses [164].

It was proposed that the elevated OPN levels in COVID-19 individuals with diabetes may increase the expression of furin, which cleaves the SARS-CoV-2 spike protein to allow virus entry into host cells [165]. The plasma levels of FL-OPN were higher in both COVID-19-infected patients with mild clinical symptoms (CV) (*p* < 0.001) and the patients with pneumonia (CP) (*p* < 0.0001) than in healthy controls (Table 1) [115]. An increase showing the same significant difference was also observed in the Ud-OPN value. ROC analysis using the data of FL-OPN, Ud-OPN, FL-Gal-9, and truncated (Tr)-Gal-9 yielded the high values to differentiate CP patients from healthy controls. ROC analysis between CV and CP patients showed that Ud-OPN had a higher AUC value (0.81) than FL-OPN (0.70), indicating that the cleaved form may reflect the pathological conditions of CP patients. Furthermore, Spearman analysis showed a significant negative association of Ud-OPN with respiratory factors and moderate positive associations with CRP, sIL-2R, ferritin, and D-dimer levels. The plasma levels of OPN were reported to be significantly higher in COVID-19-infected severe patients as compared with non-severe cases (Table 2), and the levels of OPN were associated with increased activation of macrophages, DCs, neutrophils, eosinophils, NK cells, and T and B lymphocytes in critical patients [116]. The plasma levels of FL-OPN and Ud-OPN in CP patients were significantly higher compared with those in CV patients (Table 2) [115]. These results indicate that circulating FL-OPN and Ud-OPN reflect disease severity of COVID-19.

**Figure 5:**
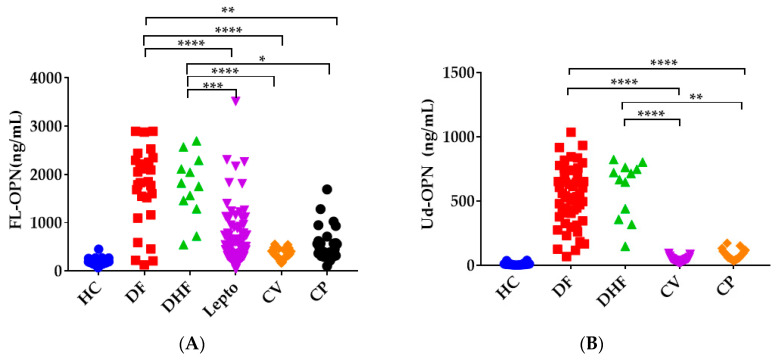
Comparative data of FL-OPN (**A**) and Ud-OPN (**B**) between dengue, leptospirosis, and COVID-19 infections. The abbreviations are DF, dengue fever; DHF, dengue hemorrhagic fever. Data are from references [9,111,115]. ****; *p* < 0.0001, ***; *p* < 0.001, **; *p* < 0.01, *; *p* < 0.05.

We explored whether the OPN levels could be different between dengue, leptospirosis, and COVID-19 (Figure 5). The levels of FL-OPN showed a wide range in DF (1833 ± 159 ng/mL), DHF (1792 ± 193 ng/mL) [11], leptospirosis (443 ± 47.3 ng/mL) [111], and CP (455 ± 67.0 ng/mL). There were significant differences between DF and CV (*p* < 0.0001) or DF and CP (*p* = 0.0059), and between DHF and CV (*p* < 0.0001) or DHF and CP (*p* = 0.011). The levels in CV and CP did not significantly differ from those in leptospirosis. The levels of Ud-OPN also varied widely in DF (540 ± 30.9 ng/mL) and DHF (692 ± 64 ng/mL) as compared with CV (42.3 ± 4.03 ng/mL) and CP (71.4 ± 6.98 ng/mL). The levels in DF were significantly different (*p* < 0.0001) from those in both CV and CP, and the levels in DHF were significantly different from those in CP (*p* = 0.0019). These findings suggest that patients with high levels of Ud-OPN are not likely to suffer from COVID-19. These findings may also explain why non-severe forms of dengue are more symptomatic than COVID-19 [164]. However, it might be early to conclude that the titers of Ud-OPN can differentiate DF, CV, or CP, because DF, DHF, and leptospirosis patients were from Manila in the Philippines, while the CV and CP patients were from Sendai, Japan. Furthermore, all patients with COVID-19 survived, indicating that their symptoms were not serious. Therefore, the aforementioned titers should be evaluated in patients from the same country in larger studies.

Some patients with COVID-19, dengue, and leptospirosis succumb to cytokine storms involving an increase of IL-6 [166]. A recent randomized trial of hospitalized patients with severe COVID-19 pneumonia reported the potential benefit of a therapy using the tocilizumab monoclonal antibody to the IL-6 receptor in the period until hospital discharge and during ICU stay [167]. Administration of tocilizumab decreased the levels of Ud-OPN, although the levels of FL-OPN did not change, indicating that the suppression of cleavage is associated with clinical amelioration [115]. The mechanisms underlying the involvement of OPN cleavage in a cytokine storm are not clear; however, these findings further suggest that the cleaved forms of OPN can be used to monitor the severity of pathological inflammation and the therapeutic effects of tocilizumab in CP patients.

It has been proposed that myeloid or tumor cell-expressed OPN acts as an immune checkpoint to suppress T-cell activation by binding to CD44 molecules on CD8 cells [168]. It is necessary to study whether the cleavage of the cell-bound OPN by MMPs leads to the activation of immune cells and causes cytokine storms. Among various inflammatory markers, MMP-9 has been strongly associated with the PO2/FiO2 ratio and can distinguish between COVID-19 patients with and without respiratory failure [169]. Furthermore, detailed analysis showed that the levels were associated with mortality of the patients [170]. It has been also linked to pleural effusions, alveolar damage, and neuroinflammation, which are often seen in COVID-19 patients, and MMP-9 inhibitors were implicated for the therapy [171]. Furthermore, a cytokine storm of COVID-19 is associated with severe coagulopathy and thrombin activation plays a key role for coagulopathy [172]. Application of thrombin inhibitors was proposed to have potential therapeutic benefits [173]. It would be necessary to study how these therapies will affect the metabolism of OPN to understand the roles of OPN in COVID-19.

## 5. Conclusions

Circulating FL-OPN is elevated in various infectious diseases that include ATL, TB, AIDS/TB, AIDS, hepatitis virus infection, dengue, leptospirosis, and COVID-19. Thrombin, MMPs, caspase 8/3, cathepsin D, plasmin, and enterokinase reportedly cleave OPN. The different functions, expression, and localization of FL-OPN and its cleaved OPNs during inflammation are noteworthy. The cleaved forms of OPN are related to the severity of TB, COVID-19, melioidosis, and trypanosomiasis. The elevations of both FL-OPN and its cleaved forms in COVID-19 are less than those in dengue or leptospirosis. The correlation of OPN levels with the severity of the disease present in the lung diseases such as TB and CP in COVID-19 and the correlation with the renal function in leptospirosis strongly suggests the levels reflect not only systemic inflammation but organ-specific inflammation. Furthermore, the levels of the cleaved forms of OPN may reflect the cytokine storm of febrile illness. New functions of truncated OPNs were analyzed and antibody therapies were proposed to prevent these functions. On the other hand, inhibition of such cleavage may also have good consequences. In any case, both basic and clinical research on this protein is expected to open a new door and create new therapies.

## Figures and Tables

**Table 1:** Comparison of OPN levels in peripheral blood among patients with infectious diseases.

Disease		OPN [ng/mL]		*p* Value	Statistic	Statistical Analysis	Sample Type	ELISA	Ref.
		Patients (*n*)	Control (*n*)						
ATL	Acute	843 (*n* = 13)	<396 (*n* = 30)	<0.0001	Median	Kruskal–Wallis test and two tail Dunn’s post test	Plasma	IBL	[100]
	Lymphoma	800 (*n* = 3)		<0.05					
	Chronic	318 (*n* = 9)		-					
	Smoldering	259 (*n* = 2)		-					
TB		600 (*n* = 48)	250 (*n* =34)	<0.0001	Median	Fisher’s test	Plasma	IBL	[104]
		159 (*n* = 37)	69 (*n* =30)	<0.0001	Median	Mann–Whitney U-test	Plasma	RDS	[105]
		666 (*n* = 49)	129 (*n* = 30)	<0.0001	Median	Kruskal–Wallis test and Mann–Whitney U-test	Plasma	IBL	[106]
		65.1 (*n* = 49)	8.34 (*n* = 30)	<0.0001				RDS	
AIDS/TB		810 (*n* = 33)	129 (*n* = 30)	<0.0001	Median	Kruskal–Wallis test and Mann–Whitney U-test	Plasma	IBL	[106]
		103 (*n* = 49)	8.34 (*n* = 30)	<0.0001				RDS	
AIDS		661 (*n* = 24)	129 (*n* = 30)	<0.0001	Median	Kruskal–Wallis test and Mann–Whitney U-test	Plasma	IBL	[106]
		123 (*n* = 24)	8.34 (*n* = 30)	<0.0001				RDS	
Hepatitis virus infection	HBV	560 * (*n* = 10)	490 * (*n* = 20)	<0.01	Mean	Student’s t-test	Serum	IBL	[107]
	ACLF	8491 (*n* = 54)	3880 (*n* = 20)	0.015	Mean	Student’s t-test	Serum	RDS	[108]
	LC	4.52 (*n* = 39)	3.23 (*n* = 14)	<0.001	Median	Mann–Whitney U-test	Plasma	RDS	[109]
	HCC	67.4 (*n* = 100)	53.7 (*n* = 194)	<0.001	Median	Student’s paired t-test	Plasma	RDS	[110]
Dengue	DF (*n* =53)	25.951	2.814 (*n* = 30)	<0.0001	Median	Dunn’s multiple comparison test	Plasma	IBL	[11]
		540	68 (*n* = 30)	<0.0001				RDS	
	DHF (*n* = 12)	27.55	2.814 (*n* = 30)	<0.0001				IBL	
		692	68 (*n* = 30)	<0.0001				RDS	
Leptospirosis		442 (*n* = 112)	187.23 (*n* = 30)	<0.0001	Median	Mann–Whitney U-test	Plasma	IBL	[111]
Melioidosis		7100 * (*n* = 33)	100 * (*n* = 31)	<0.001	Mean	Mann–Whitney U-test	Plasma	RDS	[112]
Schistosomiasis	Acute	80 * (*n* = 28)	5 ^¶^* (*n* = 21)	<0.0001	Median	Mann–Whitney U-test	Serum	RDS	[113]

OPN concentrations in the plasma or serum from patients and healthy controls are shown as the median or mean values. Abbreviations are ATL, adult T-cell leukemia; TB, tuberculosis; AIDS, acquired immunodeficiency syndrome, which is co-infected with opportunistic infections other than TB; AIDS/TB, AIDS co-infected with TB; HBV, hepatitis B virus; ACLF, acute-on-chronic liver failure; LC, liver cirrhosis; HCC, hepatocellular carcinoma; DF, dengue fever; DHF, dengue hemorrhagic fever; IBL ELISA, ELISA kit from Immuno-Biological Laboratories, used to measure plasma FL-OPN and trOPN; RDS ELISA, ELISA kit from R&D Systems, to be specific for undefined epitope and detect both FL-OPN and the cleaved forms (Ud-OPN). ^¶^ Uninfected control. * Values were read from the figure; “-”, no data.

**Table 2:** OPN levels in body fluid reflect disease severity of infectious diseases.

Disease	OPN [ng/mL]		*p* Value	Statistic	Statistical Analysis	Sample Type	ELISA	Ref.
ATL	Acute 843 (*n* = 13)	Chronic 318 (*n* = 9)	0.0002	Median	Mann–Whitney U-test	Plasma	IBL	[100]
	Acute & Lymphoma 843 (*n* = 16)	Chronic & Smoldering 318 (*n* = 11)	<0.0001					
TB	Severe PTB 192 (*n* = 10)	Non-severe PTB 114 (*n* = 10)	<0.0001	Median	Mann–Whitney U-test	Plasma	RDS	[114]
COVID-19	CP 425 (*n* = 25)	CV 330 (*n* = 23)	<0.0146	Median	Mann–Whitney U-test	Plasma	IBL	[115]
	CP 63.3 (*n* = 13)	CV 42.3 (*n* = 9)	0.0001				RDS	
	Clitically ill 13.75 (*n* = 40)	Non-critically ill 9.85 (*n* = 48)	0.002	Median	Mann–Whitney U-test	Serum	Elabscience	[116]
Melioidosis	Non-survivors 8750 * (*n* = 14)	Survivors 6200 * (*n* = 19)	<0.05	Mean	Mann–Whitney U-test	Plasma	RDS	[112]
Trypanosomiasis	Moderate neurological singns 550 * (*n* = 31)	Absence of neurological signs 100 * (*n* = 21)	<0.01	Median	Mann–Whitney U-test	CSF	RDS	[117]
	Severe neurological sings 950 * (*n* = 5)		<0.05		Kruskal–Wallis test			

OPN concentrations in body fluid are shown as the median or mean values. PTB, pulmonary TB; COVID-19, coronavirus disease 2019; CP and CV, COVID-19 infected patients with pneumonia and mild clinical symptoms, respectively, critically ill patients with COVID-19, COVID-19 patients who suffer from respiratory failure, shock, or multiorgan dysfunction; and CSF, cerebrospinal fluid. * Values were read from the figure.

## References

[1] Senger D.R., Wirth D.F., Hynes R.O. (1979). Transformed mammalian cells secrete specific proteins and phosphoproteins. Cell.

[2] Weber G.F., Cantor H. (1996). The immunology of Eta-1/osteopontin. Cytokine Growth Factor Rev..

[3] Bornstein P. (2009). Matricellular proteins: An overview. J. Cell Commun. Signal..

[4] Chiodoni C., Colombo M.P., Sangaletti S. (2010). Matricellular proteins: From homeostasis to inflammation, cancer, and metastasis. Cancer Metastasis Rev..

[5] Kon S., Maeda M., Segawa T., Hagiwara Y., Horikoshi Y., Chikuma S., Tanaka K., Rashid M.M., Inobe M., Chambers A.F. (2000). Antibodies to different peptides in osteopontin reveal complexities in the various secreted forms. J. Cell Biochem..

[6] Uede T., Katagiri Y., Iizuka J., Murakami M. (1997). Osteopontin, a coordinator of host defense system: A cytokine or an extracellular adhesive protein?. Microbiol. Immunol..

[7] Denhardt D.T., Noda M., O’Regan A.W., Pavlin D., Berman J.S. (2001). Osteopontin as a means to cope with environmental insults: Regulation of inflammation, tissue remodeling, and cell survival. J. Clin. Investig..

[8] Smith L.L., Cheung H.K., Ling L.E., Chen J., Sheppard D., Pytela R., Giachelli C.M. (1996). Osteopontin N-terminal domain contains a cryptic adhesive sequence recognized by alpha9beta1 integrin. J. Biol. Chem..

[9] Kon S., Yokosaki Y., Maeda M., Segawa T., Horikoshi Y., Tsukagoshi H., Rashid M.M., Morimoto J., Inobe M., Shijubo N. (2002). Mapping of functional epitopes of osteopontin by monoclonal antibodies raised against defined internal sequences. J. Cell Biochem..

[10] Kurata M., Okura T., Kumon Y., Tagawa M., Watanabe H., Nakahara T., Miyazaki T., Higaki J., Nose M. (2012). Plasma thrombin-cleaved osteopontin elevation after carotid artery stenting in symptomatic ischemic stroke patients. Hypertens Res..

[11] Chagan-Yasutan H., Lacuesta T.L., Ndhlovu L.C., Oguma S., Leano P.S., Telan E.F., Kubo T., Morita K., Uede T., Dimaano E.M. (2014). Elevated levels of full-length and thrombin-cleaved osteopontin during acute dengue virus infection are associated with coagulation abnormalities. Thromb Res..

[12] Maeda N., Ohashi T., Chagan-Yasutan H., Hattori T., Takahashi Y., Harigae H., Hasegawa H., Yamada Y., Fujii M., Maenaka K. (2015). Osteopontin-integrin interaction as a novel molecular target for antibody-mediated immunotherapy in adult T-cell leukemia. Retrovirology.

[13] Honda M., Kimura C., Uede T., Kon S. (2020). Neutralizing antibody against osteopontin attenuates non-alcoholic steatohepatitis in mice. J. Cell Commun. Signal..

[14] Bai G., Matsuba T., Kikuchi H., Chagan-Yasutan H., Motoda H., Ozuru R., Yamada O., Oshima Y., Hattori T. (2019). Inhibition of inflammatory-molecule synthesis in THP-1 cells stimulated with phorbol 12-myristate 13-acetate by brefelamide derivatives. Int. Immunopharm..

[15] Senger D.R., Perruzzi C.A., Gracey C.F., Papadopoulos A., Tenen D.G. (1988). Secreted phosphoproteins associated with neoplastic transformation: Close homology with plasma proteins cleaved during blood coagulation. Cancer Res..

[16] Senger D.R., Perruzzi C.A., Papadopoulos A. (1989). Elevated expression of secreted phosphoprotein I (osteopontin, 2ar) as a consequence of neoplastic transformation. Anticancer Res..

[17] Senger D.R., Perruzzi C.A., Papadopoulos A., Tenen D.G. (1989). Purification of a human milk protein closely similar to tumor-secreted phosphoproteins and osteopontin. Biochim. Biophys. Acta.

[18] Agnihotri R., Crawford H.C., Haro H., Matrisian L.M., Havrda M.C., Liaw L. (2001). Osteopontin, a novel substrate for matrix metalloproteinase-3 (stromelysin-1) and matrix metalloproteinase-7 (matrilysin). J. Biol. Chem..

[19] Dean R.A., Overall C.M. (2007). Proteomics discovery of metalloproteinase substrates in the cellular context by iTRAQ labeling reveals a diverse MMP-2 substrate degradome. Mol. Cell Proteom..

[20] Takafuji V., Forgues M., Unsworth E., Goldsmith P., Wang X.W. (2007). An osteopontin fragment is essential for tumor cell invasion in hepatocellular carcinoma. Oncogene.

[21] Kim H.J., Lee H.J., Jun J.I., Oh Y., Choi S.G., Kim H., Chung C.W., Kim I.K., Park I.S., Chae H.J. (2009). Intracellular cleavage of osteopontin by caspase-8 modulates hypoxia/reoxygenation cell death through p53. Proc. Natl. Acad. Sci. USA.

[22] Christensen B., Schack L., Kläning E., Sørensen E.S. (2010). Osteopontin is cleaved at multiple sites close to its integrin-binding motifs in milk and is a novel substrate for plasmin and cathepsin D. J. Biol. Chem..

[23] Giachelli C.M., Liaw L., Murry C.E., Schwartz S.M., Almeida M. (1995). Osteopontin expression in cardiovascular diseases. Ann. N. Y. Acad. Sci..

[24] Dvorak H.F. (1986). Tumors: Wounds that do not heal. Similarities between tumor stroma generation and wound healing. N. Engl. J. Med..

[25] Oldberg A., Franzén A., Heinegård D. (1986). Cloning and sequence analysis of rat bone sialoprotein (osteopontin) cDNA reveals an Arg-Gly-Asp cell-binding sequence. Proc. Natl. Acad. Sci. USA.

[26] Senger D.R., Perruzzi C.A., Papadopoulos-Sergiou A., Van de Water L. (1994). Adhesive properties of osteopontin: Regulation by a naturally occurring thrombin-cleavage in close proximity to the GRGDS cell-binding domain. Mol. Biol. Cell.

[27] Hu D.D., Lin E.C., Kovach N.L., Hoyer J.R., Smith J.W. (1995). A biochemical characterization of the binding of osteopontin to integrins alpha v beta 1 and alpha v beta 5. J. Biol. Chem..

[28] Miyauchi A., Alvarez J., Greenfield E.M., Teti A., Grano M., Colucci S., Zambonin-Zallone A., Ross F.P., Teitelbaum S.L., Cheresh D. (1991). Recognition of osteopontin and related peptides by an alpha v beta 3 integrin stimulates immediate cell signals in osteoclasts. J. Biol. Chem..

[29] Ross F.P., Chappel J., Alvarez J.I., Sander D., Butler W.T., Farach-Carson M.C., Mintz K.A., Robey P.G., Teitelbaum S.L., Cheresh D.A. (1993). Interactions between the bone matrix proteins osteopontin and bone sialoprotein and the osteoclast integrin alpha v beta 3 potentiate bone resorption. J. Biol. Chem..

[30] Liaw L., Almeida M., Hart C.E., Schwartz S.M., Giachelli C.M. (1994). Osteopontin promotes vascular cell adhesion and spreading and is chemotactic for smooth muscle cells in vitro. Circ. Res..

[31] Liaw L., Skinner M.P., Raines E.W., Ross R., Cheresh D.A., Schwartz S.M., Giachelli C.M. (1995). The adhesive and migratory effects of osteopontin are mediated via distinct cell surface integrins. Role of alpha v beta 3 in smooth muscle cell migration to osteopontin in vitro. J. Clin. Investig..

[32] Hu D.D., Hoyer J.R., Smith J.W. (1995). Ca2+ suppresses cell adhesion to osteopontin by attenuating binding affinity for integrin alpha v beta 3. J. Biol. Chem..

[33] Yokosaki Y., Tanaka K., Higashikawa F., Yamashita K., Eboshida A. (2005). Distinct structural requirements for binding of the integrins alphavbeta6, alphavbeta3, alphavbeta5, alpha5beta1 and alpha9beta1 to osteopontin. Matrix Biol..

[34] Barry S.T., Ludbrook S.B., Murrison E., Horgan C.M. (2000). A regulated interaction between alpha5beta1 integrin and osteopontin. Biochem. Biophys. Res. Commun..

[35] Denda S., Reichardt L.F., Müller U. (1998). Identification of osteopontin as a novel ligand for the integrin alpha8 beta1 and potential roles for this integrin-ligand interaction in kidney morphogenesis. Mol. Biol. Cell.

[36] Yokosaki Y., Kido M., Nagata N., Nikaido Y., Tsuda T., Miyake J., Manabe H. (1995). Hypoglycemia associated with localized fibrous mesothelioma of the pleura. J. UOEH.

[37] Green P.M., Ludbrook S.B., Miller D.D., Horgan C.M., Barry S.T. (2001). Structural elements of the osteopontin SVVYGLR motif important for the interaction with alpha(4) integrins. FEBS Lett..

[38] Ito N., Obata H., Saito S. (2009). Spinal microglial expression and mechanical hypersensitivity in a postoperative pain model: Comparison with a neuropathic pain model. Anesthesiology.

[39] Bayless K.J., Davis G.E. (2001). Identification of dual alpha 4beta1 integrin binding sites within a 38 amino acid domain in the N-terminal thrombin fragment of human osteopontin. J. Biol. Chem..

[40] Smith L.L., Giachelli C.M. (1998). Structural requirements for alpha 9 beta 1-mediated adhesion and migration to thrombin-cleaved osteopontin. Exp. Cell Res..

[41] Taooka Y., Chen J., Yednock T., Sheppard D. (1999). The integrin alpha9beta1 mediates adhesion to activated endothelial cells and transendothelial neutrophil migration through interaction with vascular cell adhesion molecule-1. J. Cell Biol..

[42] Yokosaki Y., Matsuura N., Sasaki T., Murakami I., Schneider H., Higashiyama S., Saitoh Y., Yamakido M., Taooka Y., Sheppard D. (1999). The integrin alpha(9)beta(1) binds to a novel recognition sequence (SVVYGLR) in the thrombin-cleaved amino-terminal fragment of osteopontin. J. Biol. Chem..

[43] Barry S.T., Ludbrook S.B., Murrison E., Horgan C.M. (2000). Analysis of the alpha4beta1 integrin-osteopontin interaction. Exp. Cell Res..

[44] Kon S., Ikesue M., Kimura C., Aoki M., Nakayama Y., Saito Y., Kurotaki D., Diao H., Matsui Y., Segawa T. (2008). Syndecan-4 protects against osteopontin-mediated acute hepatic injury by masking functional domains of osteopontin. J. Exp. Med..

[45] Morimoto J., Kon S., Matsui Y., Uede T. (2010). Osteopontin; as a target molecule for the treatment of inflammatory diseases. Curr. Drug Targets.

[46] Grassinger J., Haylock D.N., Storan M.J., Haines G.O., Williams B., Whitty G.A., Vinson A.R., Be C.L., Li S., Sørensen E.S. (2009). Thrombin-cleaved osteopontin regulates hemopoietic stem and progenitor cell functions through interactions with alpha9beta1 and alpha4beta1 integrins. Blood.

[47] Yokasaki Y., Sheppard D. (2000). Mapping of the cryptic integrin-binding site in osteopontin suggests a new mechanism by which thrombin can regulate inflammation and tissue repair. Trends Cardiovasc. Med..

[48] Senger D.R., Perruzzi C.A. (1996). Cell migration promoted by a potent GRGDS-containing thrombin-cleavage fragment of osteopontin. Biochim. Biophys. Acta.

[49] Senger D.R., Ledbetter S.R., Claffey K.P., Papadopoulos-Sergiou A., Peruzzi C.A., Detmar M. (1996). Stimulation of endothelial cell migration by vascular permeability factor/vascular endothelial growth factor through cooperative mechanisms involving the alphavbeta3 integrin, osteopontin, and thrombin. Am. J. Pathol..

[50] Desai B., Rogers M.J., Chellaiah M.A. (2007). Mechanisms of osteopontin and CD44 as metastatic principles in prostate cancer cells. Mol. Cancer.

[51] Iczkowski K.A. (2010). Cell adhesion molecule CD44: Its functional roles in prostate cancer. Am. J. Transl. Res..

[52] Weber G.F., Zawaideh S., Hikita S., Kumar V.A., Cantor H., Ashkar S. (2002). Phosphorylation-dependent interaction of osteopontin with its receptors regulates macrophage migration and activation. J. Leukoc. Biol..

[53] Katagiri Y.U., Sleeman J., Fujii H., Herrlich P., Hotta H., Tanaka K., Chikuma S., Yagita H., Okumura K., Murakami M. (1999). CD44 variants but not CD44s cooperate with beta1-containing integrins to permit cells to bind to osteopontin independently of arginine-glycine-aspartic acid, thereby stimulating cell motility and chemotaxis. Cancer Res..

[54] Shao Z., Morser J., Leung L.L.K. (2014). Thrombin cleavage of osteopontin disrupts a pro-chemotactic sequence for dendritic cells, which is compensated by the release of its pro-chemotactic C-terminal fragment. J. Biol. Chem..

[55] Myles T., Nishimura T., Yun T.H., Nagashima M., Morser J., Patterson A.J., Pearl R.G., Leung L.L. (2003). Thrombin activatable fibrinolysis inhibitor, a potential regulator of vascular inflammation. J. Biol. Chem..

[56] Sharif S.A., Du X., Myles T., Song J.J., Price E., Lee D.M., Goodman S.B., Nagashima M., Morser J., Robinson W.H. (2009). Thrombin-activatable carboxypeptidase B cleavage of osteopontin regulates neutrophil survival and synoviocyte binding in rheumatoid arthritis. Arthritis Rheum..

[57] McCawley L.J., Matrisian L.M. (2000). Matrix metalloproteinases: Multifunctional contributors to tumor progression. Mol. Med. Today.

[58] Crawford H.C., Matrisian L.M., Liaw L. (1998). Distinct roles of osteopontin in host defense activity and tumor survival during squamous cell carcinoma progression in vivo. Cancer Res..

[59] Wright J.H., McDonnell S., Portella G., Bowden G.T., Balmain A., Matrisian L.M. (1994). A switch from stromal to tumor cell expression of stromelysin-1 mRNA associated with the conversion of squamous to spindle carcinomas during mouse skin tumor progression. Mol. Carcinog..

[60] Kon S., Nakayama Y., Matsumoto N., Ito K., Kanayama M., Kimura C., Kouro H., Ashitomi D., Matsuda T., Uede T. (2014). A novel cryptic binding motif, LRSKSRSFQVSDEQY, in the C-terminal fragment of MMP-3/7-cleaved osteopontin as a novel ligand for α9β1 integrin is involved in the anti-type II collagen antibody-induced arthritis. PLoS ONE.

[61] Liaw L., Birk D.E., Ballas C.B., Whitsitt J.S., Davidson J.M., Hogan B.L. (1998). Altered wound healing in mice lacking a functional osteopontin gene (spp1). J. Clin. Investig..

[62] Wilson C.L., Heppner K.J., Rudolph L.A., Matrisian L.M. (1995). The metalloproteinase matrilysin is preferentially expressed by epithelial cells in a tissue-restricted pattern in the mouse. Mol. Biol. Cell.

[63] Sun Y., Li D., Lv X.H., Hua S.C., Han J.C., Xu F., Li X.D. (2015). Roles of osteopontin and matrix metalloproteinase-7 in occurrence, progression, and prognosis of nonsmall cell lung cancer. J. Res. Med. Sci..

[64] Leitner L., Schuch K., Jürets A., Itariu B.K., Keck M., Grablowitz V., Aszmann O.C., Prager G., Staffler G., Zeyda M. (2015). Immunological blockade of adipocyte inflammation caused by increased matrix metalloproteinase-cleaved osteopontin in obesity. Obesity.

[65] Slovacek H., Khanna R., Poredos P., Jezovnik M., Hoppensteadt D., Fareed J., Hopkinson W. (2020). Interrelationship of Osteopontin, MMP-9 and ADAMTS4 in Patients With Osteoarthritis Undergoing Total Joint Arthroplasty. Clin. Appl. Thromb. Hemost..

[66] Lindsey M.L., Zouein F.A., Tian Y., Padmanabhan Iyer R., de Castro Brás L.E. (2015). Osteopontin is proteolytically processed by matrix metalloproteinase 9. Can. J. Physiol. Pharmcol..

[67] Goncalves DaSilva A., Liaw L., Yong V.W. (2010). Cleavage of osteopontin by matrix metalloproteinase-12 modulates experimental autoimmune encephalomyelitis disease in C57BL/6 mice. Am. J. Pathol..

[68] Hwang S.M., Wilson P.D., Laskin J.D., Denhardt D.T. (1994). Age and development-related changes in osteopontin and nitric oxide synthase mRNA levels in human kidney proximal tubule epithelial cells: Contrasting responses to hypoxia and reoxygenation. J. Cell Physiol..

[69] Dewhirst M.W., Cao Y., Moeller B. (2008). Cycling hypoxia and free radicals regulate angiogenesis and radiotherapy response. Nat. Rev. Cancer.

[70] Christensen B., Kläning E., Nielsen M.S., Andersen M.H., Sørensen E.S. (2012). C-terminal modification of osteopontin inhibits interaction with the αVβ3-integrin. J. Biol. Chem..

[71] Bai G., Motoda H., Ozuru R., Chagan-Yasutan H., Hattori T., Matsuba T. (2018). Synthesis of a Cleaved Form of Osteopontin by THP-1 Cells and Its Alteration by Phorbol 12-Myristate 13-Acetate and BCG Infection. Int. J. Mol. Sci..

[72] Kohro T., Tanaka T., Murakami T., Wada Y., Aburatani H., Hamakubo T., Kodama T. (2004). A comparison of differences in the gene expression profiles of phorbol 12-myristate 13-acetate differentiated THP-1 cells and human monocyte-derived macrophage. J. Atheroscler Thromb..

[73] Fu X., Zeng L., Liu Z., Ke X., Lei L., Li G. (2016). MicroRNA-206 regulates the secretion of inflammatory cytokines and MMP9 expression by targeting TIMP3 in Mycobacterium tuberculosis-infected THP-1 human macrophages. Biochem. Biophys. Res. Commun..

[74] Rivera-Marrero C.A., Stewart J., Shafer W.M., Roman J. (2004). The down-regulation of cathepsin G in THP-1 monocytes after infection with Mycobacterium tuberculosis is associated with increased intracellular survival of bacilli. Infect. Immun..

[75] Franchini M., Mannucci P.M. (2012). Thrombin and cancer: From molecular basis to therapeutic implications. Semin. Thromb. Hemost..

[76] Yamaguchi Y., Shao Z., Sharif S., Du X.Y., Myles T., Merchant M., Harsh G., Glantz M., Recht L., Morser J. (2013). Thrombin-cleaved fragments of osteopontin are overexpressed in malignant glial tumors and provide a molecular niche with survival advantage. J. Biol. Chem..

[77] Beausoleil M.S., Schulze E.B., Goodale D., Postenka C.O., Allan A.L. (2011). Deletion of the thrombin cleavage domain of osteopontin mediates breast cancer cell adhesion, proteolytic activity, tumorgenicity, and metastasis. BMC Cancer.

[78] Hasegawa M., Segawa T., Maeda M., Yoshida T., Sudo A. (2011). Thrombin-cleaved osteopontin levels in synovial fluid correlate with disease severity of knee osteoarthritis. J. Rheumatol..

[79] Beilin O., Karussis D.M., Korczyn A.D., Gurwitz D., Aronovich R., Hantai D., Grigoriadis N., Mizrachi-Kol R., Chapman J. (2005). Increased thrombin inhibition in experimental autoimmune encephalomyelitis. J. Neurosci. Res..

[80] Davalos D., Ryu J.K., Merlini M., Baeten K.M., Le Moan N., Petersen M.A., Deerinck T.J., Smirnoff D.S., Bedard C., Hakozaki H. (2012). Fibrinogen-induced perivascular microglial clustering is required for the development of axonal damage in neuroinflammation. Nat. Commun..

[81] Davalos D., Baeten K.M., Whitney M.A., Mullins E.S., Friedman B., Olson E.S., Ryu J.K., Smirnoff D.S., Petersen M.A., Bedard C. (2014). Early detection of thrombin activity in neuroinflammatory disease. Ann. Neurol..

[82] Han M.H., Hwang S.I., Roy D.B., Lundgren D.H., Price J.V., Ousman S.S., Fernald G.H., Gerlitz B., Robinson W.H., Baranzini S.E. (2008). Proteomic analysis of active multiple sclerosis lesions reveals therapeutic targets. Nature.

[83] Boggio E., Dianzani C., Gigliotti C.L., Soluri M.F., Clemente N., Cappellano G., Toth E., Raineri D., Ferrara B., Comi C. (2016). Thrombin Cleavage of Osteopontin Modulates Its Activities in Human Cells In Vitro and Mouse Experimental Autoimmune Encephalomyelitis In Vivo. J. Immunol. Res..

[84] Cui G., Chen J., Wu Z., Huang H., Wang L., Liang Y., Zeng P., Yang J., Uede T., Diao H. (2019). Thrombin cleavage of osteopontin controls activation of hepatic stellate cells and is essential for liver fibrogenesis. J. Cell Physiol..

[85] Kitagori K., Yoshifuji H., Oku T., Sasaki C., Miyata H., Mori K.P., Nakajima T., Ohmura K., Kawabata D., Yukawa N. (2016). Cleaved Form of Osteopontin in Urine as a Clinical Marker of Lupus Nephritis. PLoS ONE.

[86] Ashkar S., Weber G.F., Panoutsakopoulou V., Sanchirico M.E., Jansson M., Zawaideh S., Rittling S.R., Denhardt D.T., Glimcher M.J., Cantor H. (2000). Eta-1 (osteopontin): An early component of type-1 (cell-mediated) immunity. Science.

[87] Koguchi Y., Kawakami K., Kon S., Segawa T., Maeda M., Uede T., Saito A. (2002). Penicillium marneffei causes osteopontin-mediated production of interleukin-12 by peripheral blood mononuclear cells. Infect. Immun..

[88] Renkl A.C., Wussler J., Ahrens T., Thoma K., Kon S., Uede T., Martin S.F., Simon J.C., Weiss J.M. (2005). Osteopontin functionally activates dendritic cells and induces their differentiation toward a Th1-polarizing phenotype. Blood.

[89] Maeno Y., Nakazawa S., Yamamoto N., Shinzato M., Nagashima S., Tanaka K., Sasaki J., Rittling S.R., Denhardt D.T., Uede T. (2006). Osteopontin participates in Th1-mediated host resistance against nonlethal malaria parasite Plasmodium chabaudi chabaudi infection in mice. Infect. Immun..

[90] Trinchieri G. (1995). The two faces of interleukin 12: A pro-inflammatory cytokine and a key immunoregulatory molecule produced by antigen-presenting cells. Ciba Found Symp..

[91] Abel B., Freigang S., Bachmann M.F., Boschert U., Kopf M. (2005). Osteopontin is not required for the development of Th1 responses and viral immunity. J. Immunol..

[92] Begum M.D., Umemura M., Kon S., Yahagi A., Hamada S., Oshiro K., Gotoh K., Nishizono A., Uede T., Matsuzaki G. (2007). Suppression of the bacterial antigen-specific T cell response and the dendritic cell migration to the lymph nodes by osteopontin. Microbiol. Immunol..

[93] Santamaría M.H., Corral R.S. (2013). Osteopontin-dependent regulation of Th1 and Th17 cytokine responses in Trypanosoma cruzi-infected C57BL/6 mice. Cytokine.

[94] Brown A., Islam T., Adams R., Nerle S., Kamara M., Eger C., Marder K., Cohen B., Schifitto G., McArthur J.C. (2011). Osteopontin enhances HIV replication and is increased in the brain and cerebrospinal fluid of HIV-infected individuals. J. Neurovirol..

[95] Iqbal J., Sarkar-Dutta M., McRae S., Ramachandran A., Kumar B., Waris G. (2018). Osteopontin Regulates Hepatitis C Virus (HCV) Replication and Assembly by Interacting with HCV Proteins and Lipid Droplets and by Binding to Receptors αVβ3 and CD44. J. Virol..

[96] Gessain A., Cassar O. (2012). Epidemiological Aspects and World Distribution of HTLV-1 Infection. Front. Microbiol..

[97] Ducasa N., Grasso D., Benencio P., Papademetrio D.L., Biglione M., Kashanchi F., Berini C., Garcia M.N. (2021). Autophagy in Human T-Cell Leukemia Virus Type 1 (HTLV-1) Induced Leukemia. Front. Oncol..

[98] Zhang J., Yamada O., Matsushita Y., Chagan-Yasutan H., Hattori T. (2010). Transactivation of human osteopontin promoter by human T-cell leukemia virus type 1-encoded Tax protein. Leuk. Res..

[99] Zhang J., Yamada O., Kida S., Matsushita Y., Yamaoka S., Chagan-Yasutan H., Hattori T. (2011). Identification of CD44 as a downstream target of noncanonical NF-κB pathway activated by human T-cell leukemia virus type 1-encoded Tax protein. Virology.

[100] Chagan-Yasutan H., Tsukasaki K., Takahashi Y., Oguma S., Harigae H., Ishii N., Zhang J., Fukumoto M., Hattori T. (2011). Involvement of osteopontin and its signaling molecule CD44 in clinicopathological features of adult T cell leukemia. Leuk. Res..

[101] Liersch R., Gerss J., Schliemann C., Bayer M., Schwöppe C., Biermann C., Appelmann I., Kessler T., Löwenberg B., Büchner T. (2012). Osteopontin is a prognostic factor for survival of acute myeloid leukemia patients. Blood.

[102] Moorman H.R., Poschel D., Klement J.D., Lu C., Redd P.S., Liu K. (2020). Osteopontin: A Key Regulator of Tumor Progression and Immunomodulation. Cancers.

[103] Nakachi S., Nakazato T., Ishikawa C., Kimura R., Mann D.A., Senba M., Masuzaki H., Mori N. (2011). Human T-cell leukemia virus type 1 tax transactivates the matrix metalloproteinase 7 gene via JunD/AP-1 signaling. Biochim. Biophys. Acta.

[104] Koguchi Y., Kawakami K., Uezu K., Fukushima K., Kon S., Maeda M., Nakamoto A., Owan I., Kuba M., Kudeken N. (2003). High plasma osteopontin level and its relationship with interleukin-12-mediated type 1 T helper cell response in tuberculosis. Am. J. Respir. Crit. Care Med..

[105] Shiratori B., Leano S., Nakajima C., Chagan-Yasutan H., Niki T., Ashino Y., Suzuki Y., Telan E., Hattori T. (2014). Elevated OPN, IP-10, and neutrophilia in loop-mediated isothermal amplification confirmed tuberculosis patients. Mediat. Inflamm..

[106] Padilla S.T., Niki T., Furushima D., Bai G., Chagan-Yasutan H., Telan E.F., Tactacan-Abrenica R.J., Maeda Y., Solante R., Hattori T. (2020). Plasma Levels of a Cleaved Form of Galectin-9 Are the Most Sensitive Biomarkers of Acquired Immune Deficiency Syndrome and Tuberculosis Coinfection. Biomolecules.

[107] Diao H., Liu X., Wu Z., Kang L., Cui G., Morimoto J., Denhardt D.T., Rittling S., Iwakura Y., Uede T. (2012). Osteopontin regulates interleukin-17 production in hepatitis. Cytokine.

[108] Liu L., Lu J., Ye C., Lin L., Zheng S., Zhang H., Lan Q., Xue Y. (2018). Serum osteopontin is a predictor of prognosis for HBV-associated acute-on-chronic liver failure. Biomed. Rep..

[109] Zhao L., Li T., Wang Y., Pan Y., Ning H., Hui X., Xie H., Wang J., Han Y., Liu Z. (2008). Elevated plasma osteopontin level is predictive of cirrhosis in patients with hepatitis B infection. Int. J. Clin. Pract..

[110] Duarte-Salles T., Misra S., Stepien M., Plymoth A., Muller D., Overvad K., Olsen A., Tjonneland A., Baglietto L., Severi G. (2016). Circulating Osteopontin and Prediction of Hepatocellular Carcinoma Development in a Large European Population. Cancer Prev. Res..

[111] Chagan-Yasutan H., Hanan F., Niki T., Bai G., Ashino Y., Egawa S., Telan E.F.O., Hattori T. (2020). Plasma Osteopontin Levels is Associated with Biochemical Markers of Kidney Injury in Patients with Leptospirosis. Diagnostics.

[112] Van der Windt G.J., Wiersinga W.J., Wieland C.W., Tjia I.C., Day N.P., Peacock S.J., Florquin S., van der Poll T. (2010). Osteopontin impairs host defense during established gram-negative sepsis caused by Burkholderia pseudomallei (melioidosis). PLoS Negl. Trop. Dis..

[113] Pereira T.A., Syn W.K., Amancio F.F., Cunha P.H., Caporali J.F., Trindade G.V., Santos E.T., Souza M.M., Andrade Z.A., Witek R.P. (2016). Osteopontin Is Upregulated in Human and Murine Acute Schistosomiasis Mansoni. PLoS Negl. Trop. Dis..

[114] Shete A., Bichare S., Pujari V., Virkar R., Thakar M., Ghate M., Patil S., Vyakarnam A., Gangakhedkar R., Bai G. (2020). Elevated Levels of Galectin-9 but Not Osteopontin in HIV and Tuberculosis Infections Indicate Their Roles in Detecting MTB Infection in HIV Infected Individuals. Front. Microbiol..

[115] Bai G., Furushima D., Niki T., Matsuba T., Maeda Y., Takahashi A., Hattori T., Ashino Y. (2021). High Levels of the Cleaved Form of Galectin-9 and Osteopontin in the Plasma Are Associated with Inflammatory Markers Tha. Int. J. Mol. Sci..

[116] Varım C., Demirci T., Cengiz H., Hacıbekiroğlu İ., Tuncer F.B., Çokluk E., Toptan H., Karabay O., Yıldırım İ. (2021). Relationship between serum osteopontin levels and the severity of COVID-19 infection. Wien. Klin. Wochenschr..

[117] Tiberti N., Hainard A., Lejon V., Robin X., Ngoyi D.M., Turck N., Matovu E., Enyaru J., Ndung’u J.M., Scherl A. (2010). Discovery and verification of osteopontin and Beta-2-microglobulin as promising markers for staging human African trypanosomiasis. Mol. Cell Proteom..

[118] Hasibuan F.M., Shiratori B., Senoputra M.A., Chagan-Yasutan H., Koesoemadinata R.C., Apriani L., Takahashi Y., Niki T., Alisjahbana B., Hattori T. (2015). Evaluation of matricellular proteins in systemic and local immune response to Mycobacterium tuberculosis infection. Microbiol. Immunol..

[119] Nau G.J., Liaw L., Chupp G.L., Berman J.S., Hogan B.L., Young R.A. (1999). Attenuated host resistance against Mycobacterium bovis BCG infection in mice lacking osteopontin. Infect. Immun..

[120] Van der Windt G.J., Wieland C.W., Wiersinga W.J., Florquin S., van der Poll T. (2009). Osteopontin is not crucial to protective immunity during murine tuberculosis. Immunology.

[121] Ridruechai C., Sakurada S., Yanai H., Yamada N., Kantipong P., Piyaworawong S., Dhepakson P., Khusmith S., Keicho N. (2011). Association between circulating full-length osteopontin and IFN-gamma with disease status of tuberculosis and response to successful treatment. Southeast Asian J. Trop. Med. Public Health.

[122] Shiratori B., Zhao J., Okumura M., Chagan-Yasutan H., Yanai H., Mizuno K., Yoshiyama T., Idei T., Ashino Y., Nakajima C. (2016). Immunological Roles of Elevated Plasma Levels of Matricellular Proteins in Japanese Patients with Pulmonary Tuberculosis. Int. J. Mol. Sci..

[123] Ragno S., Romano M., Howell S., Pappin D.J., Jenner P.J., Colston M.J. (2001). Changes in gene expression in macrophages infected with Mycobacterium tuberculosis: A combined transcriptomic and proteomic approach. Immunology.

[124] Kathamuthu G.R., Kumar N.P., Moideen K., Nair D., Banurekha V.V., Sridhar R., Baskaran D., Babu S. (2020). Matrix Metalloproteinases and Tissue Inhibitors of Metalloproteinases Are Potential Biomarkers of Pulmonary and Extra-Pulmonary Tuberculosis. Front. Immunol..

[125] Le Y., Cao W., Zhou L., Fan X., Liu Q., Liu F., Gai X., Chang C., Xiong J., Rao Y. (2020). Infection of Mycobacterium tuberculosis Promotes Both M1/M2 Polarization and MMP Production in Cigarette Smoke-Exposed Macrophages. Front. Immunol..

[126] Burdo T.H., Ellis R.J., Fox H.S. (2008). Osteopontin is increased in HIV-associated dementia. J. Infect. Dis..

[127] Chagan-Yasutan H., Saitoh H., Ashino Y., Arikawa T., Hirashima M., Li S., Usuzawa M., Oguma S., EF O.T., Obi C.L. (2009). Persistent elevation of plasma osteopontin levels in HIV patients despite highly active antiretroviral therapy. Tohoku J. Exp. Med..

[128] Richert Q., Trajtman A., Arroyave L., Toews J., Becker M., Kasper K., McLaren P., Rueda Z., Keynan Y. (2017). Systemic inflammation before and after antiretroviral therapy initiation as a predictor of immune response among HIV-infected individuals in Manitoba. Cytokine.

[129] Xing Y., Shepherd N., Lan J., Li W., Rane S., Gupta S.K., Zhang S., Dong J., Yu Q. (2017). MMPs/TIMPs imbalances in the peripheral blood and cerebrospinal fluid are associated with the pathogenesis of HIV-1-associated neurocognitive disorders. Brain Behav. Immun..

[130] Bozzelli P.L., Caccavano A., Avdoshina V., Mocchetti I., Wu J.Y., Conant K. (2020). Increased matrix metalloproteinase levels and perineuronal net proteolysis in the HIV-infected brain; relevance to altered neuronal population dynamics. Exp. Neurol..

[131] Mahmud F.J., Boucher T., Liang S., Brown A.M. (2020). Osteopontin and Integrin Mediated Modulation of Post-Synapses in HIV Envelope Glycoprotein Exposed Hippocampal Neurons. Brain Sci..

[132] Vo Quang E., Shimakawa Y., Nahon P. (2021). Epidemiological projections of viral-induced hepatocellular carcinoma in the perspective of WHO global hepatitis elimination. Liver Int..

[133] Zhang R., Pan X., Huang Z., Weber G.F., Zhang G. (2011). Osteopontin enhances the expression and activity of MMP-2 via the SDF-1/CXCR4 axis in hepatocellular carcinoma cell lines. PLoS ONE.

[134] Yang S., Wang L., Pan W., Bayer W., Thoens C., Heim K., Dittmer U., Timm J., Wang Q., Yu Q. (2019). MMP2/MMP9-mediated CD100 shedding is crucial for inducing intrahepatic anti-HBV CD8 T cell responses and HBV clearance. J. Hepatol..

[135] Sumi A., Telan E.F., Chagan-Yasutan H., Piolo M.B., Hattori T., Kobayashi N. (2017). Effect of temperature, relative humidity and rainfall on dengue fever and leptospirosis infections in Manila, the Philippines. Epidemiol. Infect..

[136] Excler J.L., Saville M., Berkley S., Kim J.H. (2021). Vaccine development for emerging infectious diseases. Nat. Med..

[137] WHO (2009). Dengue: Guidelines for Diagnosis, Treatment, Prevention and Control.

[138] WHO (1997). Dengue Haemorrhagic Fever: Diagnosis, Treatment, Prevention and Control.

[139] Kubelka C.F., Azeredo E.L., Gandini M., Oliveira-Pinto L.M., Barbosa L.S., Damasco P.V., Avila C.A., Motta-Castro A.R., Cunha R.V., Cruz O.G. (2010). Metalloproteinases are produced during dengue fever and MMP9 is associated with severity. J. Infect..

[140] Her Z., Kam Y.W., Gan V.C., Lee B., Thein T.L., Tan J.J., Lee L.K., Fink K., Lye D.C., Rénia L. (2017). Severity of Plasma Leakage Is Associated With High Levels of Interferon γ-Inducible Protein 10, Hepatocyte Growth Factor, Matrix Metalloproteinase 2 (MMP-2), and MMP-9 During Dengue Virus Infection. J. Infect. Dis..

[141] Niranjan R., Sumitha M.K., Sankari T., Muthukumaravel S., Jambulingam P. (2019). Nonstructural protein-1 (NS1) of dengue virus type-2 differentially stimulate expressions of matrix metalloproteinases in monocytes: Protective effect of paracetamol. Int. Immunopharmacol..

[142] Chagan-Yasutan H., Ndhlovu L.C., Lacuesta T.L., Kubo T., Leano P.S., Niki T., Oguma S., Morita K., Chew G.M., Barbour J.D. (2013). Galectin-9 plasma levels reflect adverse hematological and immunological features in acute dengue virus infection. J. Clin. Virol..

[143] WHO (2003). Human Leptospirosis: Guidance for Diagnosis, Surveillance and Control.

[144] Torgerson P.R., Hagan J.E., Costa F., Calcagno J., Kane M., Martinez-Silveira M.S., Goris M.G., Stein C., Ko A.I., Abela-Ridder B. (2015). Global Burden of Leptospirosis: Estimated in Terms of Disability Adjusted Life Years. PLoS Negl. Trop. Dis..

[145] Chagan-Yasutan H., Chen Y., Lacuesta T.L., Leano P.S., Iwasaki H., Hanan F., Taurustiati D., Ohmoto Y., Ashino Y., Saitoh H. (2016). Urine Levels of Defensin α1 Reflect Kidney Injury in Leptospirosis Patients. Int. J. Mol. Sci..

[146] Vijayachari P., Sugunan A.P., Shriram A.N. (2008). Leptospirosis: An emerging global public health problem. J. Biosci..

[147] Becquart P., Wauquier N., Nkoghe D., Ndjoyi-Mbiguino A., Padilla C., Souris M., Leroy E.M. (2010). Acute dengue virus 2 infection in Gabonese patients is associated with an early innate immune response, including strong interferon alpha production. BMC Infect. Dis..

[148] Iwasaki-Hozumi H., Chagan-Yasutan H., Ashino Y., Hattori T. (2021). Blood Levels of Galectin-9, an Immuno-Regulating Molecule, Reflect the Severity for the Acute and Chronic Infectious Diseases. Biomolecules.

[149] Cagliero J., Villanueva S., Matsui M. (2018). Leptospirosis Pathophysiology: Into the Storm of Cytokines. Front. Cell Infect. Microbiol..

[150] Cheng A.C., Currie B.J. (2005). Melioidosis: Epidemiology, pathophysiology, and management. Clin. Microbiol. Rev..

[151] Gassiep I., Armstrong M., Norton R. (2020). Human Melioidosis. Clin. Microbiol. Rev..

[152] Conejero L., Potempa K., Graham C.M., Spink N., Blankley S., Salguero F.J., Pankla-Sranujit R., Khaenam P., Banchereau J.F., Pascual V. (2015). The Blood Transcriptome of Experimental Melioidosis Reflects Disease Severity and Shows Considerable Similarity with the Human Disease. J. Immunol..

[153] Kasozi K.I., Zirintunda G., Ssempijja F., Buyinza B., Alzahrani K.J., Matama K., Nakimbugwe H.N., Alkazmi L., Onanyang D., Bogere P. (2021). Epidemiology of Trypanosomiasis in Wildlife-Implications for Humans at the Wildlife Interface in Africa. Front. Vet. Sci..

[154] Hainard A., Tiberti N., Robin X., Ngoyi D.M., Matovu E., Enyaru J.C., Müller M., Turck N., Ndung’u J.M., Lejon V. (2011). Matrix metalloproteinase-9 and intercellular adhesion molecule 1 are powerful staging markers for human African trypanosomiasis. Trop. Med. Int. Health.

[155] Chitsulo L., Engels D., Montresor A., Savioli L. (2000). The global status of schistosomiasis and its control. Acta Trop..

[156] Tanaka H., Tsuji M. (1997). From discovery to eradication of schistosomiasis in Japan: 1847–1996. Int. J. Parasitol..

[157] Pereira T.A., Syn W.K., Machado M.V., Vidigal P.V., Resende V., Voieta I., Xie G., Otoni A., Souza M.M., Santos E.T. (2015). Schistosome-induced cholangiocyte proliferation and osteopontin secretion correlate with fibrosis and portal hypertension in human and murine schistosomiasis mansoni. Clin. Sci..

[158] Chuah C., Jones M.K., Burke M.L., McManus D.P., Owen H.C., Gobert G.N. (2014). Defining a pro-inflammatory neutrophil phenotype in response to schistosome eggs. Cell Microbiol..

[159] Wiersinga W.J., Rhodes A., Cheng A.C., Peacock S.J., Prescott H.C. (2020). Pathophysiology, Transmission, Diagnosis, and Treatment of Coronavirus Disease 2019 (COVID-19): A Review. JAMA.

[160] Yan G., Lee C.K., Lam L.T.M., Yan B., Chua Y.X., Lim A.Y.N., Phang K.F., Kew G.S., Teng H., Ngai C.H. (2020). Covert COVID-19 and false-positive dengue serology in Singapore. Lancet Infect. Dis..

[161] Silvestre O.M., Costa L.R., Lopes B.V.R., Barbosa M.R., Botelho K.K.P., Albuquerque K.L.C., Souza A.G.S., Coelho L.A., de Oliveira A.J., Barantini C.B. (2020). Previous dengue infection and mortality in COVID-19. Clin. Infect. Dis..

[162] Nicolete V.C., Rodrigues P.T., Johansen I.C., Corder R.M., Tonini J., Cardoso M.A., de Jesus J.G., Claro I.M., Faria N.R., Sabino E.C. (2021). Interacting Epidemics in Amazonian Brazil: Prior Dengue Infection Associated with Increased COVID-19 Risk in a Population-Based Cohort Study. Clin. Infect. Dis..

[163] Niriella M.A., Ediriweera D.S., De Silva A.P., Premarathna B.H.R., Jayasinghe S., de Silva H.J. (2021). Dengue and leptospirosis infection during the coronavirus 2019 outbreak in Sri Lanka. Trans. R. Soc. Trop. Med. Hyg..

[164] Joubert A., Andry F., Bertolotti A., Accot F., Koumar Y., Legrand F., Poubeau P., Manaquin R., Gerardin P., Levin C. (2021). Distinguishing non severe cases of dengue from COVID-19 in the context of co-epidemics: A cohort study in a SARS-CoV-2 testing center on Reunion island. PLoS Negl. Trop. Dis..

[165] Adu-Agyeiwaah Y., Grant M.B., Obukhov A.G. (2020). The Potential Role of Osteopontin and Furin in Worsening Disease Outcomes in COVID-19 Patients with Pre-Existing Diabetes. Cells.

[166] Fajgenbaum D.C., June C.H. (2020). Cytokine Storm. N. Engl. J. Med..

[167] Rosas I.O., Bräu N., Waters M., Go R.C., Hunter B.D., Bhagani S., Skiest D., Aziz M.S., Cooper N., Douglas I.S. (2021). Tocilizumab in Hospitalized Patients with Severe Covid-19 Pneumonia. N. Engl. J. Med..

[168] Klement J.D., Paschall A.V., Redd P.S., Ibrahim M.L., Lu C., Yang D., Celis E., Abrams S.I., Ozato K., Liu K. (2018). An osteopontin/CD44 immune checkpoint controls CD8+ T cell activation and tumor immune evasion. J. Clin. Investig..

[169] Ueland T., Holter J.C., Holten A.R., Müller K.E., Lind A., Bekken G.K., Dudman S., Aukrust P., Dyrhol-Riise A.M., Heggelund L. (2020). Distinct and early increase in circulating MMP-9 in COVID-19 patients with respiratory failure. J. Infect..

[170] Abers M.S., Delmonte O.M., Ricotta E.E., Fintzi J., Fink D.L., de Jesus A.A.A., Zarember K.A., Alehashemi S., Oikonomou V., Desai J.V. (2021). An immune-based biomarker signature is associated with mortality in COVID-19 patients. JCI Insight.

[171] Stukalov A., Girault V., Grass V., Karayel O., Bergant V., Urban C., Haas D.A., Huang Y., Oubraham L., Wang A. (2021). Multilevel proteomics reveals host perturbations by SARS-CoV-2 and SARS-CoV. Nature.

[172] Ranucci M., Sitzia C., Baryshnikova E., Di Dedda U., Cardani R., Martelli F., Corsi Romanelli M. (2020). Covid-19-Associated Coagulopathy: Biomarkers of Thrombin Generation and Fibrinolysis Leading the Outcome. J. Clin. Med..

[173] Aliter K.F., Al-Horani R.A. (2021). Thrombin Inhibition by Argatroban: Potential Therapeutic Benefits in COVID-19. Cardiovasc. Drugs Ther..

